# Toward high‐efficiency photovoltaics‐assisted electrochemical and photoelectrochemical CO_2_ reduction: Strategy and challenge

**DOI:** 10.1002/EXP.20230001

**Published:** 2023-07-10

**Authors:** Jin Hyuk Cho, Joonhee Ma, Soo Young Kim

**Affiliations:** ^1^ Department of Materials Science and Engineering Korea University Seoul Republic of Korea

**Keywords:** catalysts, electrochemical CO_2_ reduction reaction, photoelectrochemical CO_2_ photovoltaic cell, reduction reaction

## Abstract

The realization of a complete techno‐economy through a significant carbon dioxide (CO_2_) reduction in the atmosphere has been explored to promote a low‐carbon economy in various ways. CO_2_ reduction reactions (CO_2_RRs) can be induced using sustainable energy, including electric and solar energy, using systems such as electrochemical (EC) CO_2_RR and photoelectrochemical (PEC) systems. This study summarizes various fabrication strategies for non‐noble metal, copper‐based, and metal–organic framework‐based catalysts with excellent Faradaic efficiency (FE) for target carbon compounds, and for noble metals with low overvoltage. Although EC and PEC systems achieve high energy conversion efficiency with excellent catalysts, they still require external power and lack complete bias–free operation. Therefore, photovoltaics, which can overcome the limitations of these systems, have been introduced. The utilization of silicon and perovskite‐based solar cells for photovoltaics‐assisted EC (PV‐EC) and photovoltaics‐assisted PEC (PV‐PEC) CO_2_RR systems are cost‐efficient, and the III–V semiconductor photoabsorbers achieved high solar‐to‐carbon efficiency. This work focuses on PV‐EC and PV‐PEC CO_2_RR systems and their components and then summarizes the special cell configurations, including the tandem and stacked structures. Additionally, the study discusses current issues, such as low energy conversion, expensive PV, theoretical limits, and industrial scale–up, along with proposed solutions.

## Graphical abstract

This study explores methodologies for establishing a low–carbon economy by reducing atmospheric CO_2_ through sustainable electrochemical and photoelectrochemical CO_2_ reduction reactions. It presents diverse fabrication approaches for efficient catalysts.To surmount the limitations of these systems, photovoltaic systems are introduced, and the study highlights contemporary impediments and potential remedies, encompassing inadequate energy conversion rates, exorbitant expenses, and industrial practicability.

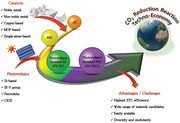

## 1 INTRODUCTION

**SCHEME 1:**
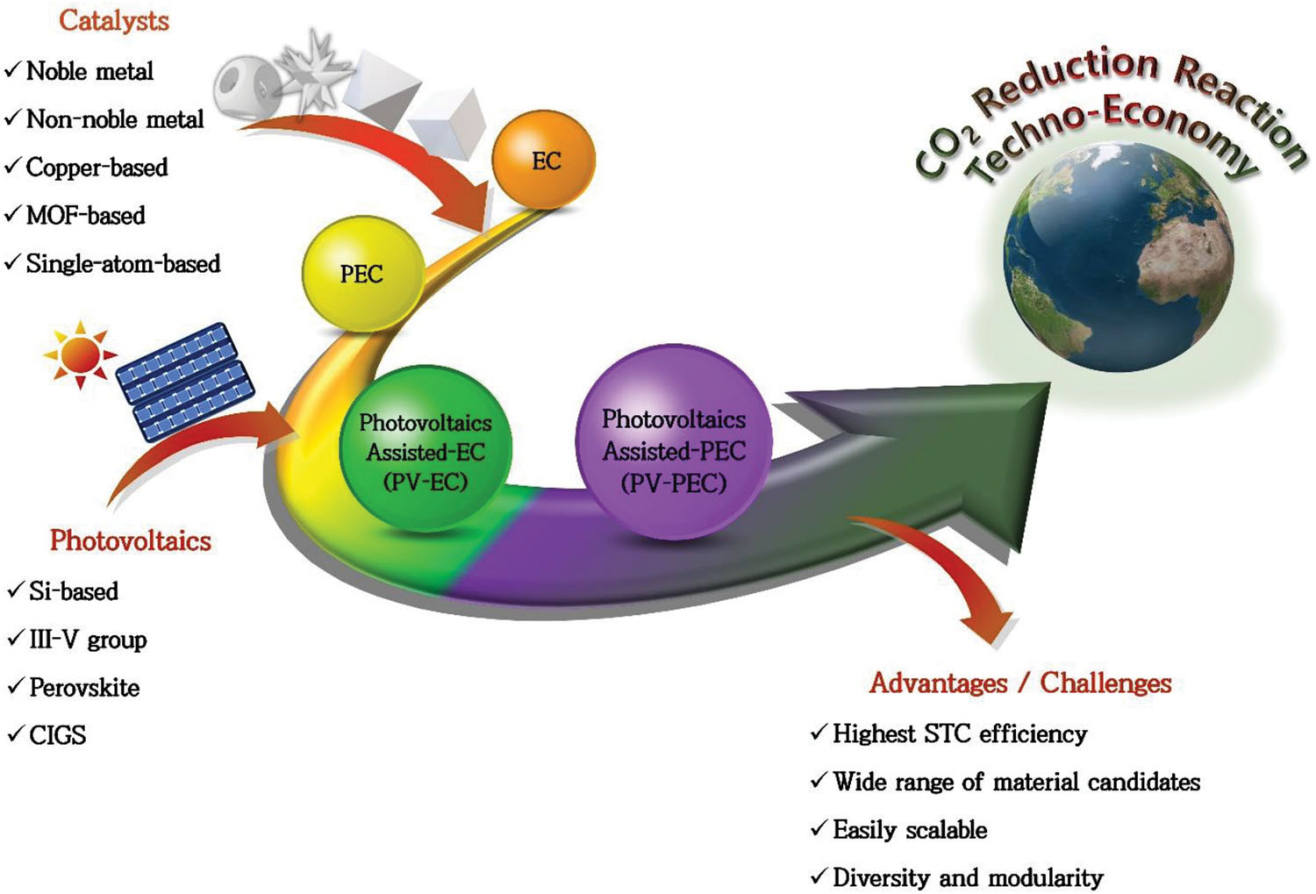
Illustration of advances in the CO_2_RR systems for techno‐economy.

Since the 19th century, the acceleration of industrial development has led to a substantial increase in the combustion of fossil fuels.^[^ ^1^ ^]^ The excessive emission of carbon dioxide (CO_2_) generated during the burning of fossil fuels accumulates in the atmosphere and traps greenhouse gases that cause not only abnormal climates such as cyclones, floods, and droughts but also anthropological problems.^[^ ^2^, ^3^ ^]^ Therefore, there is an urgent need to find a variety of sustainable and renewable energy, driven in part by technological advancements, to replace fossil fuels.^[^ ^4^, ^5^ ^]^ Currently, in response to the challenges posed by climate change, electrochemical (EC) and photoelectrochemical (PEC) reactions with the hydrogen evolution reaction (HER) and CO_2_ reduction reaction (CO_2_RR) are considered efficient potential solutions owing to their high energy efficiency and cost‐effectiveness.^[^ ^6^, ^7^, ^8^, ^9^, ^10^, ^11^ ^]^ CO_2_RR not only reduces the concentration of CO_2_ in the atmosphere but also produces additional value‐added carbon compounds.^[^ ^4^, ^12^, ^13^ ^]^ However, CO_2_ molecules are extremely inert gases in the atmosphere owing to the thermochemically stable activity of the C═O bond; therefore, it is imperative to utilize efficient catalysts.^[^ ^14^, ^15^ ^]^ Moreover, photovoltaics (PV), using sustainable solar energy, is capable of serving additional power to the CO_2_RR system in combination with EC and PEC systems, which are known as photovoltaic‐electrochemical (PV‐EC) and photovoltaic‐photoelectrochemical (PV‐PEC) systems, respectively.^[^ ^16^ ^]^ These systems require 2.6 V to carry out the oxygen evolution reaction (OER) at the anode and CO_2_RR at the cathode, which can be supplied by a solar cell.^[^ ^17^ ^]^ Therefore, designing efficient OER and CO_2_RR catalysts, as well as innovative PV device systems, is essential for achieving high solar‐to‐carbon (STC) energy conversion efficiency.^[^ ^18^, ^19^ ^]^ As depicted in Scheme 1, we organized advances in the PV‐assisted CO_2_RR for techno‐economy. To ensure the effective development and implementation of CO_2_ technologies, it is necessary to conduct a life cycle assessment (LCA) and techno‐economic analysis (TEA). LCA evaluates the environmental impact of a technology or product throughout its lifecycle, aiming to reduce its negative effects.^[^ ^20^ ^]^ TEA assesses the economic feasibility of technology development and application by analyzing its costs and benefits.^[^ ^21^ ^]^ Therefore, considering both LCA and TEA is crucial in determining the direction of technology development in the context of carbon dioxide technologies.

In this review, we discuss the current challenges faced by PV‐EC and PV‐PEC cell systems, including theoretical limitations arising from the inherent semiconductor bandgap, inhomogeneous uniformity due to increased active area, and efficiency degradation. In particular, we focus on EC and PEC catalysts because carbon compounds are manipulated during CO_2_RR through sequential electron and charge transfer,^[^ ^22^ ^]^ which are determined by the free Gibbs energy of CO_2_ absorption and the desorption energy of the products at the interface of the CO_2_ molecules and the catalyst electrode.^[^ ^19^, ^23^, ^24^ ^]^ Although noble metals are not competitive in terms of industry, gold (Au) and silver (Ag) have many advantages, including low overpotential and high Faradaic efficiency (FE) over a wide range of potentials for the formation of CO from CO_2_,^[^ ^25^, ^26^, ^27^ ^]^ and can serve as cathode components of PV‐EC and PV‐PEC because of these advantages. In addition, there is growing interest in non‐noble catalysts, including metal–organic framework (MOF)‐based and single‐atom‐based catalysts,^[^ ^28^, ^29^, ^30^ ^]^ which are considered state‐of‐the‐art due to their abundant reserves, unique morphology, reconstruction, superior electron‐charge transfer, excellent utilization, and tunable band gap energy.^[^ ^31^ ^]^ Furthermore, only copper (Cu) is feasible among the diverse elements to produce C_2+_ carbon compounds via continuous C─C coupling under CO_2_RR.^[^ ^32^, ^33^ ^]^ Therefore, we briefly explain intensive efforts to attain high selectivity for the desired carbon compounds by manipulating the surface facets of Cu, restructuring the morphology of Cu nanostructures, and causing synergistic effects from heterogeneous catalysts.^[^ ^34^, ^35^, ^36^ ^]^ To achieve a complete techno‐economy, PV provides new possibilities to convert STC efficiency with minimal energy consumption by constructing self‐contained PV‐integrated CO_2_RR reactors.^[^ ^37^ ^]^ Therefore, we introduce the performance of the PV‐EC and PV‐PEC type of CO_2_RR system that combines various types of PV including Si‐based,^[^ ^38^ ^]^ perovskite‐based,^[^ ^39^ ^]^ and GaAs‐based^[^ ^40^ ^]^ as well as tandem structures, by evaluating the current/current density–voltage (*I/J–V*) characteristics.^[^ ^41^ ^]^

## 2 ELECTROCHEMICAL CO_2_RR

Among all the CO_2_ conversion methods, the electrochemical reduction of CO_2_ stands out due to its advantages, including an easy‐to‐operate system and a controllable process.^[^ ^42^, ^43^ ^]^ Several strategies have been employed to enhance the performance of electrochemical CO_2_ reduction, such as designing and optimizing electrocatalysts^[^ ^44^, ^45^ ^]^ and developing integrated strategies for CO_2_RR electrolyzer system engineering.^[^ ^46^ ^]^ Currently, various types of electrocatalysts, such as metals,^[^ ^47^, ^48^ ^]^ metal oxides,^[^ ^49^ ^]^ alloys,^[^ ^50^ ^]^ and single‐atom catalysts (SAC),^[^ ^51^ ^]^ have been studied to improve the CO_2_RR performance. Electrochemical catalysts are classified into four groups: noble metals, non‐noble metals, Cu‐based, and single‐atom catalysts.

### 2.1 Noble metal

**FIGURE 1:**
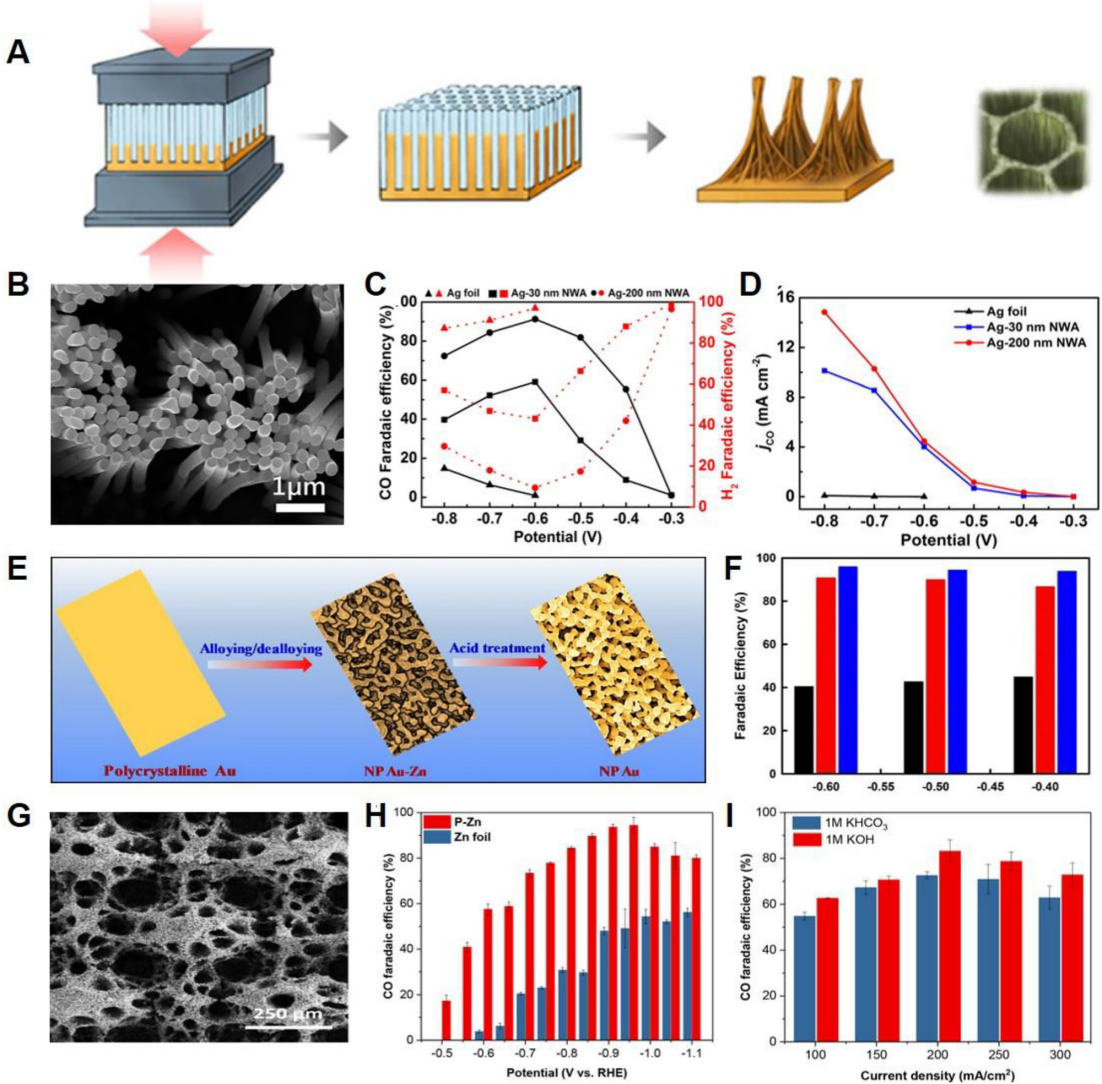
Electrochemical performances of noble‐based and non‐noble‐based catalysts for CO_2_RR. (A) Schematic of the process of synthesis of Ag NWAs. (B) SEM image of Ag NWA. (C) CO Faradaic efficiencies. (D) Partial CO current density at different applied potentials. Reproduced with permission.^[^ ^53^ ^]^ Copyright 2018, American Chemical Society. (E) Scheme illustration of the fabrication of the NP Au. (F) FE_CO_ of Au, Np Au–Zn, and NP Au. Reproduced with permission.^[^ ^55^ ^]^ Copyright 2018, Elsevier. (G) SEM image of P‐Zn electrocatalyst. (H) FE_CO_ of P‐Zn and Zn foil. (I) FE_CO_ of P‐Zn in different electrolytes. Reproduced with permission.^[^ ^60^ ^]^ Copyright 2019, American Chemical Society.

Noble metals such as Au, Ag, and Pd have been found to demonstrate remarkable activity and selectivity in catalyzing the electrochemical reduction of CO_2_, particularly in the conversion of CO_2_ to CO. This is mainly due to their capability in stabilizing the *COOH intermediate, a crucial intermediate species in the CO_2_RR process, as well as their weak adsorption affinity towards the ^∗^CO intermediate. Ag nanocoral catalysts were fabricated by Hsieh et al. utilizing an oxidation‐reduction procedure with chloride anions to promote the intrinsic electrocatalytic activity of bulk Ag.^[^ ^52^ ^]^ As a result, the Ag nanocoral catalyst demonstrated a high CO_2_RR performance with FE for CO (FE_CO_) of 95% at a low overpotential of 0.37 V versus reversible hydrogen electrode (RHE), and high long‐term stability for 72 h at −0.6 V versus RHE. The results indicate that the existence of chloride anions plays a crucial part in the enhancement of CO_2_ reduction performance. Vertically standing Ag nanowire arrays (NWAs) with outstanding electrocatalytic performance have been reported using an easy nanomolding method (Figure 1A).^[^ ^53^ ^]^ This facile method increases the electrical conductivity of electrocatalysts and facilitates the charge transfer process, leading to a more efficient CO_2_RR. Two different types of anode aluminum oxide (AAO) templates were used to fabricate Ag‐200 nm and Ag‐30 nm NWAs. As shown in Figure 1B, the as‐synthesized Ag‐200 nm NWA exhibited nanoarray morphologies. It was observed that Ag‐200 nm NWAs showed superior CO performance, requiring only a small onset potential of ≈200 mV, maximum FE_CO_ of 91 % at −0.6 V versus RHE, and CO current density (*j*_CO_) of up to ≈5 mA cm^−2^ (Figure 1C,D).

Zhu et al. fabricated ultrathin Au nanowires (NWs) using a seed‐mediated growth method for reducing CO_2_ to CO.^[^ ^54^ ^]^ Electrochemical measurements indicated that the Au NWs demonstrated high activity and selectivity toward CO with an onset potential of −0.2 V versus RHE, FE up to 94% at −0.35 V versus RHE, and great stability of 6 h at −0.35 V versus RHE in the CO_2_‐saturated 0.5 m KHCO_3_. Chen et al. demonstrated facile EC alloying/dealloying of polycrystalline Au with a combination of ZnCl_2_ and ethylene glycol for the fabrication of 3D nanoporous Au electrocatalysts (Figure 1E).^[^ ^55^ ^]^ Further acid treatment with H_2_SO_4_ followed by calcination at 150°C, resulted in the creation of new active sites, which led to an enhanced catalytic performance. The EC measurements indicate that the nanoporous Au exhibited excellent electrocatalyst performance toward CO with an FE up to 96 % at −0.6 V versus RHE in a CO_2_‐saturated 0.1 m NaHCO_3_ solution (Figure 1F). Shao et al. proposed twisted Pd–Au NWs, featuring a distinctive core–shell, using a simple template‐free approach.^[^ ^56^ ^]^ The optimized Pd–Au NWs displayed outstanding performance compared with Pd nanoparticles with an FE of 94% for CO at −0.6 V versus RHE, and a low overpotential of 90 mV. The synthesis of monodisperse Au nanoparticles was carried out by Mistry et al. utilizing the inverse micelle encapsulation approach, followed by the complete removal of ligands using O_2_ plasma treatment.^[^ ^57^ ^]^ To confirm the size‐dependent catalytic activity of electrocatalysts, a series of catalysts ranging in size from 1 to 8 nm were synthesized. The significant enhancement in activity observed for nanoparticles below 2 nm, as determined through DFT calculations, can be attributed to the increased concentration of low‐coordinated sites. Cao et al. reported a molecular surface functionalization method for modulating gold nanoparticle (Au NP) electrocatalysts for the reduction of CO_2_ to CO.^[^ ^58^ ^]^ The Au NP catalyst functionalized with N‐heterocyclic carbene showed an impressive enhancement in its FE of 83% for CO reduction in aqueous solution, with an overpotential of ≈0.46 V, and a remarkable 7.6‐fold rise in current density compared to that of the unmodified Au NP. Lu et al. successfully fabricated a nanoporous Ag electrocatalyst, aimed at circumventing the high overpotential necessity of the conventional polycrystalline Ag electrocatalyst.^[^ ^59^ ^]^ A two‐step dealloying process of an Ag–Al precursor led to the production of the nanoporous np‐Ag catalyst. The technique involved selective etching of Al, which resulted in the reorganization of the remaining Ag atoms into a 3D interconnected nanoporous structure. As‐synthesized Ag electrocatalyst exhibited nearly 100% CO FE, 8 h of long‐term stability, and a Tafel slope of 58 mV dec^−1^.

### 2.2 Non‐noble metal

Among non‐noble metals, transition metals such as Ni, Zn, and Fe are the most popular.

Luo et al. electrodeposited Zn^2+^ on Cu mesh to fabricate highly porous Zn catalysts.^[^ ^60^ ^]^ Scanning electron microscopy (SEM) images of the electrodeposited Zn catalysts revealed that the corresponding electrocatalysts had highly porous structures, resulting in superior performance (Figure 1G). Specifically, Zn electrocatalysts exhibit FE_CO_ of 95% and a current density of 27 mA cm^−2^ at −0.95 V versus RHE in H‐type cell (Figure 1H), and 84% FE_CO_ and current density of 200 mA cm^−2^ in a flow cell (Figure 1I). These outstanding performances can be attributed to the structural porosity of P‐Zn, which increases the active site density and enhances the local pH effect, further surpassing the hydrogen evolution reaction (HER). Jiang et al. fabricated surface‐regulated Ni nanoparticles which are supported on N‐doped CMK‐3 using a pyrolysis method in Ar atmosphere.^[^ ^61^ ^]^ By coordinating with N and O, the as‐synthesized electrocatalyst exhibits electronic properties different from metallic Ni, resulting in an exceptional FE_CO_ of nearly 100%, a high CO partial current density of 13 mA cm^−2^, and a turnover frequency of 4.25 s^−1^. Li et al. demonstrated a PCN‐222(Fe)/CNTs catalyst by loading PCN‐222(Fe) onto CNTs through an in situ solvothermal process, yielding an unprecedented performance for CO_2_‐to‐CO reduction.^[^ ^62^ ^]^ Due to the synergistic effect between PCN‐222(Fe) and CNTs, the electrocatalyst exhibited exceptional electrocatalytic performance with a FE_CO_ of 95.5%, TOF of 448.76 h^−1^, and high durability over 10 h at −0.6 V versus RHE.

Metal–organic frameworks (MOF) are promising nanomaterials for the CO_2_RR because of their large specific surface area, adjustable porosity, and composition.^[^ ^63^ ^]^ Wang et al. used zeolitic imidazolate frameworks (ZIFs), a class of MOFs that have a similar topology with zeolites, as catalysts for the electrochemical reduction of CO_2_.^[^ ^64^ ^]^ Three different ZIF‐8 catalysts were synthesized using various Zn sources: ZnSO_4_, Zn(NO_3_)_2_, and Zn(AC)_2_. EC measurements demonstrated that ZIF‐8 prepared with ZnSO_4_, showed excellent CO selectivity under a wide potential range from −1.5 to −1.9 V versus SCE, reaching a maximum FE of 65.5% at −1.8 V versus SCE. Similarly, ultrasmall ZIF‐8s were fabricated using a facile sol–gel method by Zhou et al., and SEM images revealed that the as‐prepared ZIF‐8 exhibited an orthododecahedral structure with a size of 80 nm.^[^ ^65^ ^]^ Ultrasmall ZIF−8 electrocatalysts display FE_CO_ of ≈90 % at −1.5 V versus RHE, a partial current density of ≈5 mA cm^−2^, and long‐term stability of 12.5 h at −1.8 V versus RHE.

Various metals, including Bi, In, and Sn are considered efficient electrocatalysts for the conversion of CO_2_ to formate (HCOO^−^). Among them, Bismuth has attracted much attention in CO_2_RR owing to its large HER overpotential and its ability to strongly bind to *OCHO species.

**FIGURE 2:**
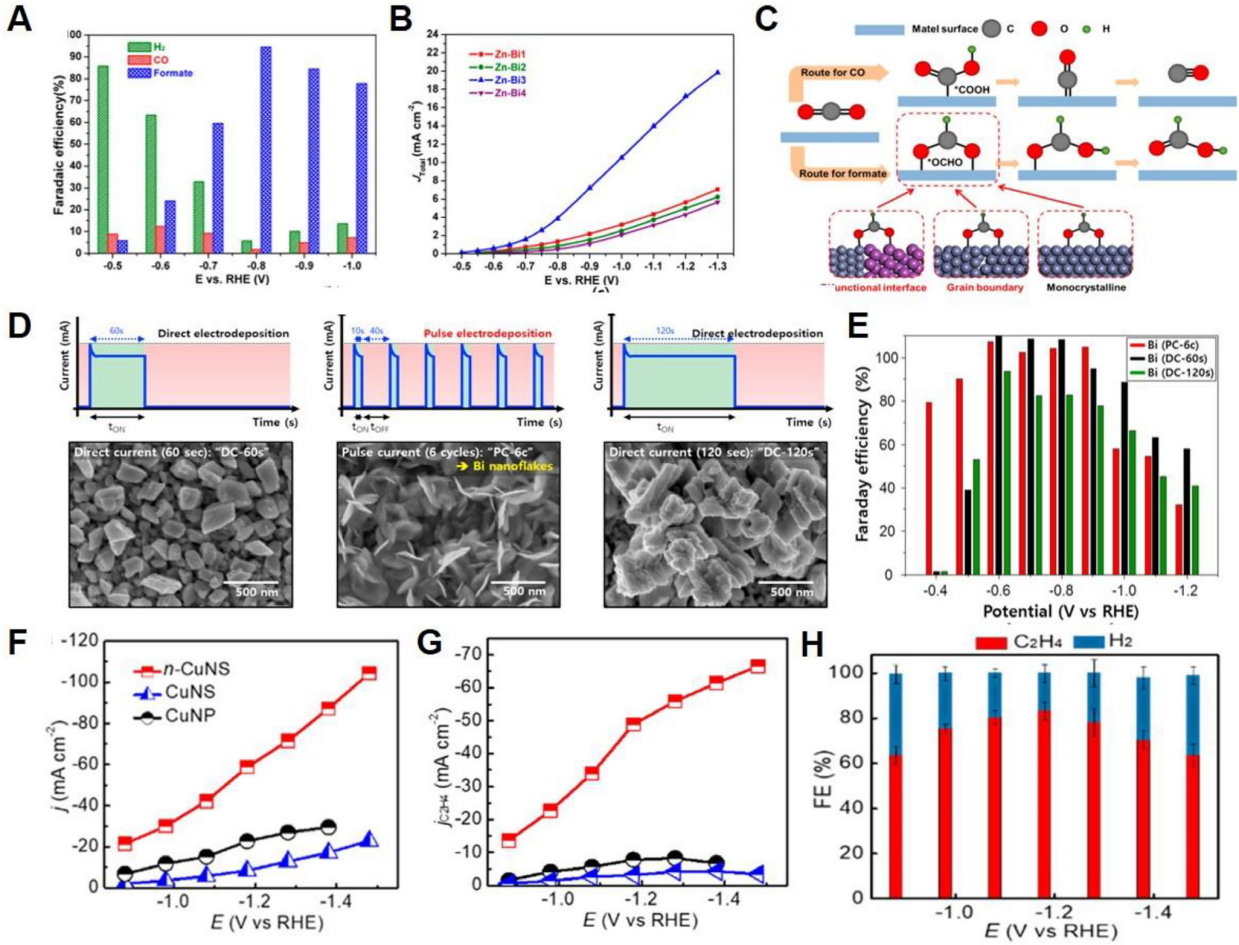
Electrochemical performances of non‐noble‐based and Cu‐based‐catalysts for CO_2_RR. (A) FE and (B) current density of all different catalysts. (C) CO reduction pathways for CO and formate formation. Reproduced with permission.^[^ ^66^ ^]^ Copyright 2019, American Chemical Society. (D) Schematics of the preparation of the Bi(PC‐6c), Bi(DC‐60s), and Bi(DC‐120s). (E) HCOO^–^ FE at different potentials. Reproduced with permission.^[^ ^67^ ^]^ Copyright 2017, Elsevier. (F) Total current density and (G) partial C_2_H_4_ current densities of n‐CuNS, CuNS, and CuNP. (H) FEs of n‐CuNS for C_2_H_4_ and H_2_ production. Reproduced with permission.^[^ ^72^ ^]^ Copyright 2020, American Chemical Society.

For example, Zhang et al. fabricated a high‐performance Bi–Zn bimetallic catalyst by surface modification of a Zn catalyst through a hydrothermal procedure with different concentrations of Bi(NO_3_)_3_ solution.^[^ ^66^ ^]^ The optimized Bi–Zn bimetallic catalyst showed exceptional performance toward formate with FE of 94%, a current density of 4 mA cm^−2^, and long‐term stability of 7 h under −0.8 V versus RHE with CO production of less than 10% over the entire potential area (Figure 2A,B). Bifunctional interfaces between the bimetal and grain boundaries were attributed to the superb selectivity toward formate by favoring *OCHO intermediate adsorption on the catalyst surface (Figure 2C). Lee et al. demonstrated a novel approach for the construction of Bi nanoflakes using the pulse‐electrodeposition method for the conversion of CO_2_ to formate.^[^ ^67^ ^]^ Various Bi nanostructures have been obtained using different electrodeposition methods. As shown in Figure 2D, nanodot‐shaped Bi particles and dendrite‐shaped Bi were obtained using DC‐60s and DC‐120s, respectively, and Bi nanoflakes were synthesized using pulse deposition for six cycles (PC‐6c).^[^ ^68^ ^]^ The Bi nanoflakes electrocatalysts exhibited exceptional CO_2_RR performance with formate FE of 79.5% at −0.4 V versus RHE and achieved a maximum FE of nearly 100% at −0.6 V versus RHE (Figure 2E). Moreover, Bi nanoflakes showed 10 h of great long‐term durability at −0.8 V versus RHE without significant decay of FE_CO_. Recently, nanoporous bismuth (np‐Bi) with a 3D ligament‐channel network structure was synthesized using a chemical dealloying approach. Mg_92_Bi_8_ consisting of Mg and Mg_3_Bi_2_ was selected as the precursor, and Mg was completely dissolved in tartaric acid, while Bi maintained stability during the process. Consequently, np‐Bi exhibited an outstanding formate FE of 94% at −0.9 V versus RHE with a maximum partial current density of 62 mA cm^−2^ at −1.2 V versus RHE, and 500 mA cm^−2^ at a low overpotential of 420 mV, in H‐type cell and flow cell, respectively.

### 2.3 Copper‐based catalyst for C_2+_ product

Cu is a unique metal for its exceptional capacity to effectively convert CO_2_ into hydrocarbons such as methane (CH_4_) and ethylene (C_2_H_4_), owing to its ability to moderately adsorb the *CO intermediate.^[^ ^47^, ^69^, ^70^ ^]^ Because the electrocatalytic performance of Cu electrodes is usually determined by the Cu crystal facets, shape control of Cu nanoparticles (NPs) has been widely studied for desirable CO_2_RR selectivity.^[^ ^71^ ^]^ Zhang et al. synthesized nanodefective Cu nanosheets using an electrochemical reduction method.^[^ ^72^ ^]^ Cu nanosheet electrocatalysts with defects exhibited a better total current density, maximum ethylene FE of 83% at −1.18 V versus RHE without CO generation, and partial current density of 66.5 mA cm^−2^ at −1.48 V versus RHE, which is much higher than those of its counterparts (Figure 2F–H). Such nanodefective structures promote ethylene production by enhancing the adsorption of intermediates and hydroxyl ions on the electrocatalyst.

Recently, simple approaches have been reported for fabricating ultrathin CuO nanoplate arrays through anodic oxidation for the conversion of CO_2_ to C_2_H_4_.^[^ ^73^ ^]^ Benefitting from stable Cu/Cu_2_O interfaces, the catalyst exhibits remarkable C_2_H_4_ FE of 84.5%, partial current densities of 92.5 mA cm^−2^ at −0.81 V versus RHE, and high stability for 55 h. Yang et al. prepared 1D ultrathin fivefold twinned Cu NWs for the conversion of CO_2_ to methane.^[^ ^74^ ^]^ Corresponding SEM and transmission electron microscopy (TEM) images revealed ultrathin NW structures with diameters of ≈20 nm. Owing to its abundant edge sites, Cu NWs showed outstanding CH_4_ selectivity with a maximum FE_CH4_ up to 55% at −1.25 V versus RHE and selectivity.

### 2.4 Single‐atom catalyst

SACs feature isolated metal atoms anchored on a support material as active sites.^[^ ^51^, ^75^ ^]^ Unlike traditional catalysts, such as nanoparticles/clusters, SACs can maximize the efficiency of metal atom utilization by almost 100%, resulting in greater exposure of active sites, further leading to outstanding catalytic activity, excellent product selectivity, and stability.^[^ ^75^, ^76^, ^77^ ^]^ Among the various types of metals, Ni‐, Fe‐, and Co‐based SACs have been proven to exhibit outstanding CO_2_RR performance. Li et al. synthesized Ni SACs with Ni–N_4_ active sites using a topochemical transformation method for converting CO_2_ to CO.^[^ ^78^ ^]^ This strategy prevents Ni atoms from agglomerating, providing abundant active sites and consequently enhancing the CO_2_RR performance. The as‐prepared electrocatalyst demonstrates remarkable FE_CO_ over 90% across a wide potential range from −0.5 to −0.9 V versus RHE and reached an FE_CO_ of ≈100% at −0.81 V versus RHE with a current density of 29 mA cm^−2^.

**FIGURE 3:**
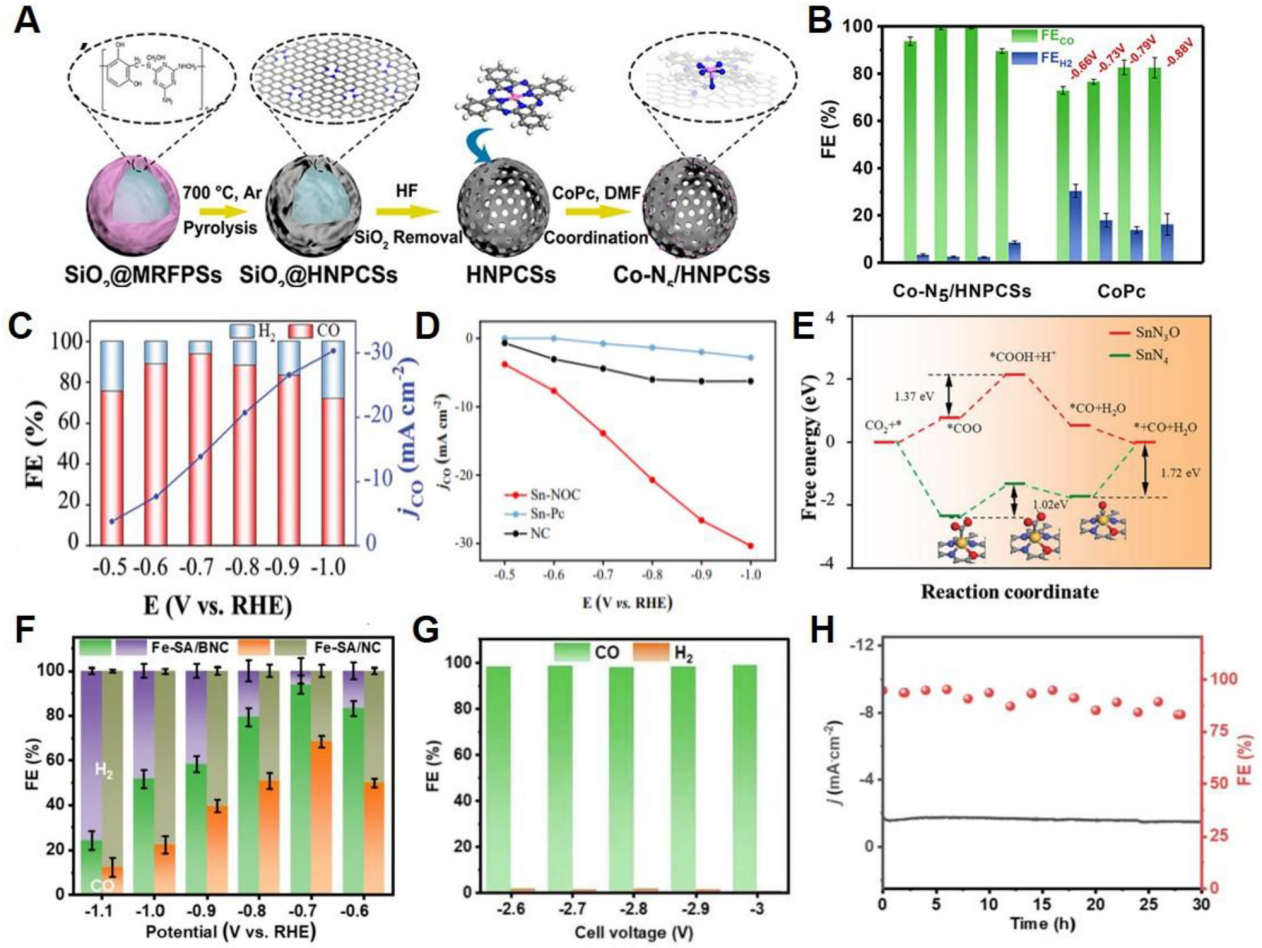
Electrochemical performances of SACs for CO_2_RR. (A) Schematic of the preparation of Co–N_5_/HNPCSs. (B) CO FEs. Reproduced with permission.^[^ ^80^ ^]^ Copyright 2018, American Chemical Society. (C) CO FEs and partial CO current densities of Sn‐NOC. (D) CO partial current densities of SN‐NOC, Sn‐PC, and NC. (E) Free energy diagram for electrochemical CO_2_ reduction to CO on Sn_3_O and SnN_4_. Reproduced with permission.^[^ ^83^ ^]^ Copyright 2021, John Wiley and Sons. (F) FE_CO_ of Fe‐SA/BNC using H‐cell and (G) using MEA. (H) Electrochemical stability test at −0.7 V versus RHE. Reproduced with permission.^[^ ^88^ ^]^ Copyright 2022, Elsevier.

Ni SACs with coordinatively unsaturated Ni–N active sites were fabricated by high‐temperature calcination of Zn/Ni bimetallic ZIF‐8.^[^ ^79^ ^]^ As prepared Ni SACs electrocatalyst showed FE of CO higher than 90% over a wide potential range (−0.53 to −1.03 V versus RHE), the current density of up to 71.5 ± 2.9 mA cm^−2^, and exceptional TOF of 10,087 ± 216 h^−1^ at −1.03 V versus RHE. Density functional theory (DFT) calculations indicated that such a coordinatively unsaturated Ni–N site contributed to enhancing the CO_2_RR performance, outperforming the HER. Pan et al. fabricated an efficient CO_2_RR electrocatalyst with Co sites atomically dispersed on polymer‐derived hollow N‐doped porous carbon spheres (HNPCSs) (Figure 3A).^[^ ^80^ ^]^ Field‐emission scanning electron microscopy (FE‐SEM) and high‐resolution TEM images of the HNPCSs indicated a uniform hollow spherical structure. Owing to their large surface area, abundant active sites, and high electrical conductivity, HNPCSs demonstrated high CO_2_RR performance toward CO with FE above 90% within the full potential range and reached a maximum FE of 99.4% at −0.79 V versus RHE (Figure 3B). Xin et al. synthesized Zn single atoms anchored onto microporous N‐doped carbon (SA‐Zn/MNC) using dissolution and carbonization methods for the CO_2_RR to CH_4_.^[^ ^81^ ^]^ Owing to its conductivity and highly exposed active sites, as‐prepared SA‐Zn/MNC showed FE_CH4_ of % at −1.8 V versus SCE, the partial current density of CO production of −32 mA cm^−2^, and outstanding long‐term stability for 35 h. Recently, Wu et al. produced atomically dispersed Fe atoms coordinated to N (Fe─N) within carbon nanorods (Fe─N─C) through high‐temperature pyrolysis of a 3D sea urchin‐like FeOOH‐polyaniline composite.^[^ ^82^ ^]^ Owing to its highly porous structure with abundant exposed active sites, as well as its large specific surface area, the optimized Fe─N─C electrocatalyst exhibited a high FE_CO_ of 95 % at a small overpotential of 530 mV with j_CO_ of 1.9 mA cm^−2^. Guao et al. fabricated Sn SACs with atomically dispersed SnN_3_O_1_ active sites embedded in an N‐rich carbon matrix for an efficient EC conversion of CO_2_ to CO.^[^ ^83^ ^]^ Unlike the Sn─N_4_ configuration, asymmetric SnN_3_O_1_ configurations have been found to show better performance for the conversion of CO_2_ to CO with an FE of more than 90%, CO partial current density of 14 mA cm^−2^ at −0.7 V versus RHE, and extraordinary TOF of 23,340.5 h^−1^ (Figure 3C,D). DFT calculations demonstrated that the unique SnN_3_O_1_ configuration of the Sn SACs electrocatalysts decreased the activation energies required to form *CO and *COOH, further facilitating CO formation (Figure 3E). To develop high‐performance SACs electrocatalysts, heteroatoms such as S,^[^ ^84^ ^]^ B,^[^ ^85^ ^]^ and P^[^ ^86^ ^]^ were introduced to alter the coordination environment of the center atoms and electronic structures.^[^ ^87^ ^]^ Liu et al. developed single atomic Fe electrocatalysts anchored on B/N co‐doped carbon supports using ferroceneboronic acid (FBA) for doping Fe and B into ZIF‐8 with a one‐to‐one atomic ratio of Fe and B.^[^ ^88^ ^]^ FBA@ZIF‐8 was first synthesized and Fe‐SA/BNC was subsequently obtained via high‐temperature pyrolysis at 900°C for 2 h. The Fe‐SA/BNC exhibited outstanding CO_2_RR performance with an FE_CO_ of ≈94% at −0.7 V versus RHE, a current density of ≈25 mA cm^−2^, and remarkable long‐term stability of 30 h using H‐cell and FE_CO_ of 99%, the current density of 130 mA cm^−2^ using membrane electrode assembly (Figure 3F−H). These electrochemical test results emphasize the importance of introducing boron into Fe‐SA/NC. The diverse electrocatalysts used for the CO_2_RR are summarized in Table 1.

**TABLE 1:** Summary of electrocatalysts for CO_2_RR.

Type	Catalyst	Electrolyte	Product	FE (%)	Ref.
Noble metals	Ag nanocorals	0.1 m KHCO_3_	CO	95	^[^ ^52^ ^]^
	Ag‐200 nm NWA	0.5 m KHCO_3_	CO	91	^[^ ^53^ ^]^
	Ag NWs	0.5 m KHCO_3_	CO	94	^[^ ^54^ ^]^
	NP Au	0.1 m NaHCO_3_	CO	95.86	^[^ ^55^ ^]^
	Pd–Au NWs	0.5 m KHCO_3_	CO	94.3	^[^ ^56^ ^]^
	Au NPs	0.1 m KHCO_3_	CO	45	^[^ ^57^ ^]^
	Au–Cb NP	0.1 m KHCO_3_	CO	83	^[^ ^58^ ^]^
	np‐Ag	0.5 m KHCO_3_	CO	92	^[^ ^59^ ^]^
Non‐noble metals	P‐Zn	0.1 m KHCO_3_	CO	95	^[^ ^60^ ^]^
	N,O‐Ni/CMK3	0.5 m KHCO_3_	CO	97	^[^ ^61^ ^]^
	PCN‐222(Fe)	0.5 m KHCO_3_	CO	95.5	^[^ ^62^ ^]^
	ZIF‐8^SO4^	0.5 m NaCl	CO	65.5	^[^ ^64^ ^]^
	ZIF‐880 nm	0.5 m KHCO_3_	CO	90	^[^ ^65^ ^]^
	Bi‐modified Zn	0.5 m NaHCO_3_	HCOO^−^	94	^[^ ^66^ ^]^
	Bi nanoflakes	0.1 m KHCO_3_	HCOO^−^	100	^[^ ^67^ ^]^
	Np–Bi	0.5 m KHCO_3_	HCOO^−^	94	^[^ ^68^ ^]^
Cu‐based	n‐CuNS	0.1 m KHCO_3_	C_2_H_4_	83.2	^[^ ^72^ ^]^
	R‐CuO‐NPs	0.5 m KCl	C_2_H_4_	84.5	^[^ ^73^ ^]^
	Cu NW	0.1 m KHCO_3_	CH_4_	55	^[^ ^74^ ^]^
Single‐atom catalysts	Ni–N_4_–C	0.5 m KHCO_3_	CO	99	^[^ ^78^ ^]^
	C–Zn_1_Ni_4_ ZIF‐8	0.1 m KHCO_3_	CO	98	^[^ ^79^ ^]^
	Co–N_5_/HNPCSs	0.2 m NaHCO_3_	CO	99.4	^[^ ^80^ ^]^
	SA‐Zn/MNC	1 m KHCO_3_	CO	85	^[^ ^81^ ^]^
	Fe–N–C‐0.5	0.5 m KHCO_3_	CO	95	^[^ ^82^ ^]^
	Sn‐NOC	0.1 m KHCO_3_	CO	94	^[^ ^83^ ^]^
	Fe‐SA/BNC	0.1 m KHCO_3_	CO	94	^[^ ^84^ ^]^

## 3 PHOTOELECTROCATALYTIC CO_2_ REDUCTION

Photoelectrochemical (PEC) CO_2_ reduction possesses the advantages of both photocatalytic and electrocatalytic CO_2_ reduction and has attracted a great deal of interest.^[^ ^89^, ^90^, ^91^ ^]^

PEC CO_2_RR proceeds through the following steps:^[^ ^90^ ^]^

1. Generation of electron–hole pairs.
2. Charge separation/transport by an external bias.
3. Surface redox reactions

A large amount of electron–hole recombination occurs at each stage, releasing energy as light or heat. Therefore, the separation and transfer of photogenerated carriers in semiconductors are key factors for effectively improving solar conversion efficiency.

Improving the performance of photocathodes has been the subject of extensive research and development. In addition to doping^[^ ^92^, ^93^ ^]^ and nanostructuring,^[^ ^94^, ^95^, ^96^, ^97^, ^98^ ^]^ coupling a co‐catalyst^[^ ^91^ ^]^ with a light‐absorbing semiconductor has emerged as one of the most effective approaches for enhancing PEC performance. Normally, bare semiconductor surfaces are inert and can barely activate CO_2_ molecules, leading to poor PEC CO_2_RR performance. To solve these problems, various types of co‐catalysts have been loaded on the photoelectrode to reduce the activation energy for CO_2_ reduction and suppress surface charge recombination, thus accelerating the surface reaction kinetics.

### 3.1 Noble metal

**FIGURE 4:**
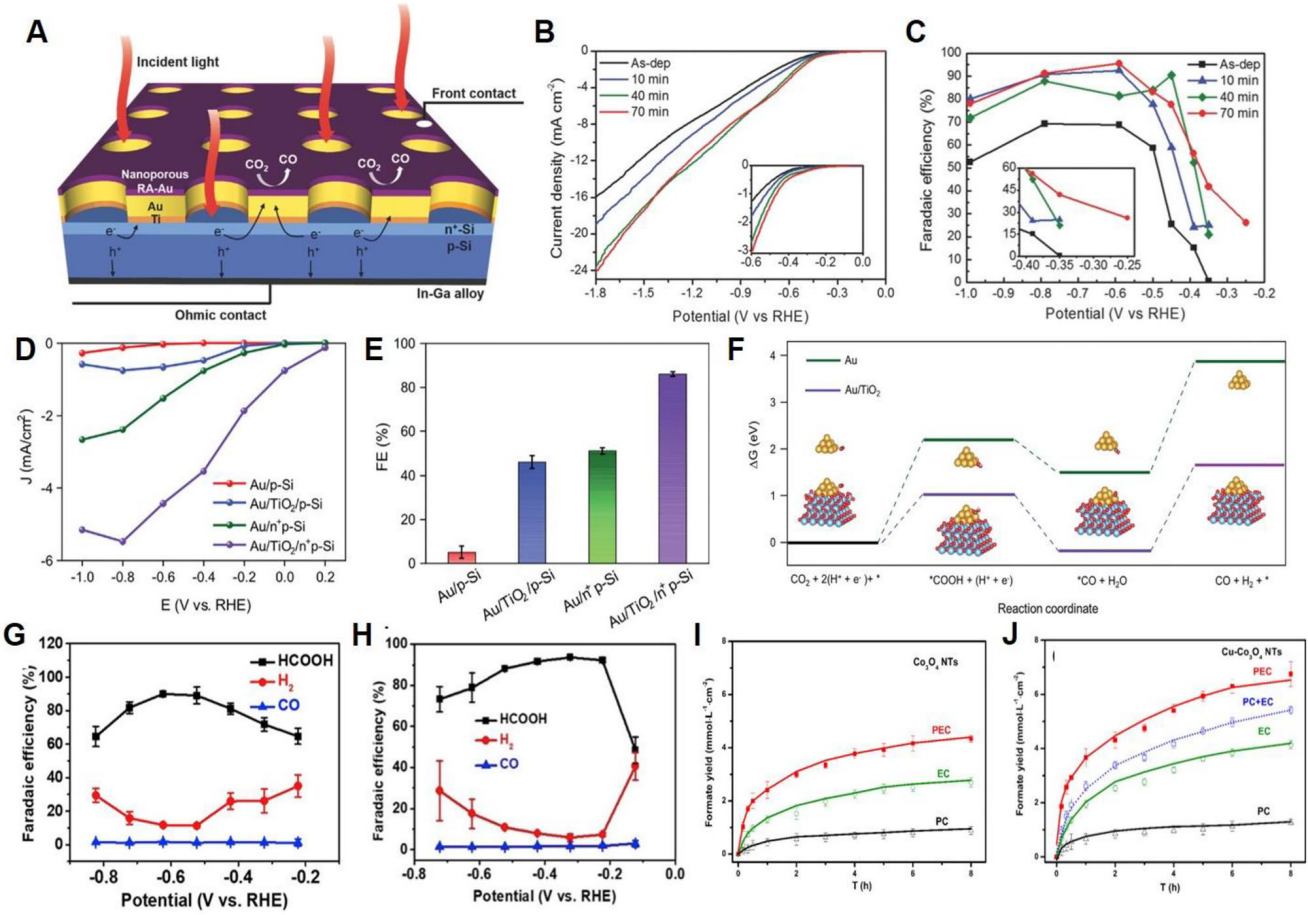
Electrochemical performances of co‐catalysts for CO_2_ reduction. (A) The fabrication process of Nanoporous Au thin films on Si Photoelectrodes. (B) Total current densities of Au thin films. (C) CO FEs at various potentials with different RA treatment times. Reproduced with permission.^[^ ^99^ ^]^ Copyright 2016, John Wiley and Sons. (D) LSV curve and (E) FE_CO_ of Au/p^–^Si, Au/TiO_2_/p^–^Si, Au/n^+^p^–^Si, and Au/TiO_2_/n^+^p^–^Si. (F) Free energy diagram of CO_2_RR over Au and Au/TiO_2_, respectively. Reproduced with permission.^[^ ^101^ ^]^ Copyright 2022, John Wiley and Sons. (G) FEs of formate, H_2_, and CO on Si/Bi‐15 min. (H) Faradaic efficiencies for formate, H_2_, and CO on p‐Si/Bi. Reproduced with permission.^[^ ^104^ ^]^ Copyright 2019, Materials Today Chemistry. (I) Formate yield on Co_3_O_4_ NTs electrode. (J) Formate yield on Cu‐Co_3_O_4_ NTs electrode. Reproduced with permission.^[^ ^107^ ^]^ Copyright 2015, American Chemical Society.

Noble metals, including Au, Ag, Pt, Pd, Ru, and Rh, are known to be the most active co‐catalyst for PEC CO_2_ reduction owing to their high catalytic activity and selectivity. Song et al. demonstrated a nanoporous mesh‐type Au thin film co‐catalyst loaded onto a Si photocathode via mild electrochemical oxidation and reduction of the Au thin film (Figure 4A).^[^ ^99^ ^]^ As shown in Figure 4B, the current density and onset potentials of all RA‐Au thin films were significantly improved compared with those of the untreated Au thin film. The optimized co‐catalyst showed outstanding performance toward PEC reaction for the reduction of CO_2_ to CO with an FE of up to 91% at the CO_2_/CO equilibrium potential of −0.11 V versus RHE in an aqueous solution under 1 sun illumination (Figure 4C). To design an efficient semiconductor/co‐catalyst interface, Jang et al. prepared a ZnTe/ZnO photocathode with Au nanoparticles deposited using an e‐beam evaporator.^[^ ^100^ ^]^ By loading Au NPs onto a photocathode, a Schottky junction was formed at the interface between the Au NPs and ZnTe, resulting in the improved separation of photogenerated carriers and electron transfer into the electrolyte. As a result, Au‐coupled ZnTe/ZnO‐NW photocathode delivered outstanding PEC performance with a photocurrent density of −16.0 mA cm^−2^ and incident photon‐to‐current conversion efficiency of 97% compared with those of a bare electrode (−7.9 mA cm^−2^, 68%). Recently, Wang et al. developed a novel strategy for producing highly efficient PEC photocathodes by coupling plasmonic Au NPs and n^+^p^−^Si through a TiO_2_ interlayer.^[^ ^101^ ^]^ The Au/TiO_2_/n^+^p^−^ Si photocathode produced 86% FE_CO_ with a partial current density of −5.52 mA cm^−2^ at −0.8 V versus RHE (Figure 4D,E). DFT calculations indicated that the synergistic effect of layering Au and TiO_2_ facilitated *COOH formation and *CO desorption, thereby promoting the conversion of CO_2_ to CO (Figure 4F). An innovative method of controlled chemical etching on Si wafers using etching solutions containing Ag^+^ ions was reported to synthesize an Si surface uniformly deposited with an Ag particulate film.^[^ ^102^ ^]^ The PEC performance of the method was excellent with a large photocurrent density of ≈10 mA cm^−2^ under 0.5 sun, outstanding FE of ≈90% at 0.5 V versus RHE for CO, and excellent stability of 8 h.

### 3.2 Non‐noble metal

Despite the prominent PEC CO_2_RR of noble metal‐based co‐catalysts, their high cost and limited availability restricted their further large‐scale CO_2_ conversion. Hence, the establishment of inexpensive catalysts with sufficiently high activity and stability is essential. Bi‐ and Sn‐based co‐catalysts are well known for their particularly high selectivity for the reduction of CO_2_ to formate (HCOOH). Choi et al. prepared heterojunction Sn‐coupled p‐Si NWAs using an Ag‐catalyzed electroless chemical etching method.^[^ ^103^ ^]^ HR‐TEM and HAADF‐STEM measurements indicated that the Sn nanoparticles were uniformly distributed on the wire array. These heterojunction wire/Sn arrays show extraordinary PEC performance toward HCOOH, compared with planar p‐Si and wire arrays with FE of 40% and 88% in single‐cell and H‐type cells, respectively. Recently, Ding et al. prepared Si/Bi photocathodes with an enhanced interface through the Bi^3+^‐assisted chemical etching of Si wafers and assessed their PEC CO_2_ reduction performance.^[^ ^104^ ^]^ The optimized Si/Bi photocathodes exhibit outstanding catalytic activity, with a positive onset potential, large photocurrent density of 10 mA cm^−2^ under 0.5 sun, and excellent formate FE of >90% (Figure 4G). Moreover, the photocurrent density was improved up to 12 mA cm^−2^ when the Si surface was exposed using the photolithography method (Figure 4H). Ma et al. demonstrated p‐type Si nanowire arrays (SiNWs) loaded with a core–shell‐structured Ni@In co‐catalyst for the reduction of CO_2_ to formate.^[^ ^105^ ^]^ P‐type SiNWs were synthesized via a metal‐assisted chemical etching method, and Ni@In/SiNWs photocathodes were subsequently fabricated by a photodeposition approach. As a result, compared with pristine SiNWs, Ni@In/SiNWs catalyst exhibited superior performance for CO_2_ to HCOOH conversion with a formation rate of 58 μmol h^−1^ cm^−2^ along with high FE of 87% at −1.2 V versus RHE.

### 3.3 Copper‐based

Deng et al. synthesized a Cu_2_O photocathode coated with a metal–organic framework (MOF) material, Cu_3_(BTC)_2_, to protect the Cu_2_O from unwanted photocorrosion, enhance charge separation and electron transfer to active sites, and offer numerous active sites for catalytic CO_2_ reduction.^[^ ^106^ ^]^ The obtained Cu_3_(BTC)_2_/Cu_2_O photocathode with adequate active sites exhibited a maximum CO selectivity of up to 95% and 0.83% of STC efficiency at −2.07 V versus Fc/Fc^+^, surpassing those of a bare Cu_2_O electrode. Shen et al. reported a Cu‐decorated Co_3_O nanotube electrode for PEC CO_2_ reduction to formate.^[^ ^107^ ^]^ Co_3_O_4_ NTs were first constructed on Co foil by anodization, and Cu electrodeposition was subsequently performed to synthesize metallic Cu‐loaded Co_3_O_4_ nanotube array electrodes. Because Cu NPs are positively charged due to their interaction with Co_3_O_4_, CO_2_ adsorption on Cu NPs occurs in the form of O‐Cu, promoting the protonation of the carbon atom, and resulting in the formation of formate. This synergistic effect between Co_3_O_4_ NTs and metallic Cu NPs results in remarkable PEC CO_2_ reduction performance with nearly 100% selectivity and a maximum production rate of 6.75 mmol·L^−1^·cm^−2^ in 8 h PEC process, which is superior to Co_3_O_4_ NTs without metallic Cu NPs (Figure 4I,J). Table 2 summarizes the PEC‐CO_2_RR performance of the co‐catalysts.

**TABLE 2:** Summary of co‐catalysts for PEC CO_2_ reduction.

Photocathode	Co‐catalyst	Electrolyte	Product	FE (%)	Ref.
pn^+^‐Si	RA‐Au film	0.2 m KHCO_3_	CO	91	^[^ ^99^ ^]^
ZnTe/ZnO‐nanowire	Au NPs	0.5 m KHCO_3_	CO	97	^[^ ^100^ ^]^
TiO_2_/n^+^p^−^ Si	Au NPs	0.1 m KHCO_3_	CO	86	^[^ ^101^ ^]^
p‐Si photocathode	Particulate Ag film	0.5 m KHCO_3_	CO	90	^[^ ^102^ ^]^
p‐Si nanowire	Sn NPs	0.1 m KHCO_3_	HCOO^−^	88	^[^ ^103^ ^]^
p‐Si photocathode	Bi film	0.5 m KHCO_3_	HCOO^−^	90	^[^ ^104^ ^]^
p‐Si nanowire arrays	Ni@In core–shell	0.1 m KHCO_3_	HCOO^−^	87	^[^ ^105^ ^]^
Cu_2_O photocathode	Cu_3_(BTC)_2_	4 m NaOH	CO	95	^[^ ^106^ ^]^
Co_3_O_4_ NTs arrays	Cu NPs	0.1 m Na_2_SO_4_	HCOO^−^	Production rate of 6.75 mmol·L^−1^·cm^−2^	^[^ ^107^ ^]^

## 4 PHOTOVOLTAIC‐POWERED ELECTROCATALYTIC CO_2_RR REACTOR

PV‐EC offers a sustainable and renewable approach to carbon reduction by utilizing renewable energy sources, such as solar power, to drive the electrochemical conversion of CO_2_. This not only reduces reliance on non‐renewable energy sources but also helps mitigate the impact of climate change. Additionally, the technology has the potential to offer a reliable and scalable approach to reducing carbon emissions while producing valuable chemical products and enabling the storage of renewable energy. Despite advances in PV‐EC technology, the integration of PV and EC systems still presents challenges in achieving optimal charge transfer efficiency and minimizing losses at their interface, and the performance of the combined system can vary greatly depending on the approach taken to address these issues and the specific characteristics of the PV and EC components.

### 4.1 C_1_ product

In most cathode electrodes of the PV‐EC CO_2_RR combination, CO_2_RR and HER are dominant while the oxygen evolution reaction is dominant at the anode electrode.^[^ ^108^, ^109^ ^]^ As described above, noble metal catalysts, including Au and Ag, with low overpotentials capable of suppressing hydrogen formation as well as active CO formation from CO_2_ have been utilized as electrocatalyst cathodes. The PV‐EC CO_2_RR characteristics of the catalyst were confirmed by FE, current density, and overpotential measurements.^[^ ^79^, ^109^, ^110^ ^]^

**FIGURE 5:**
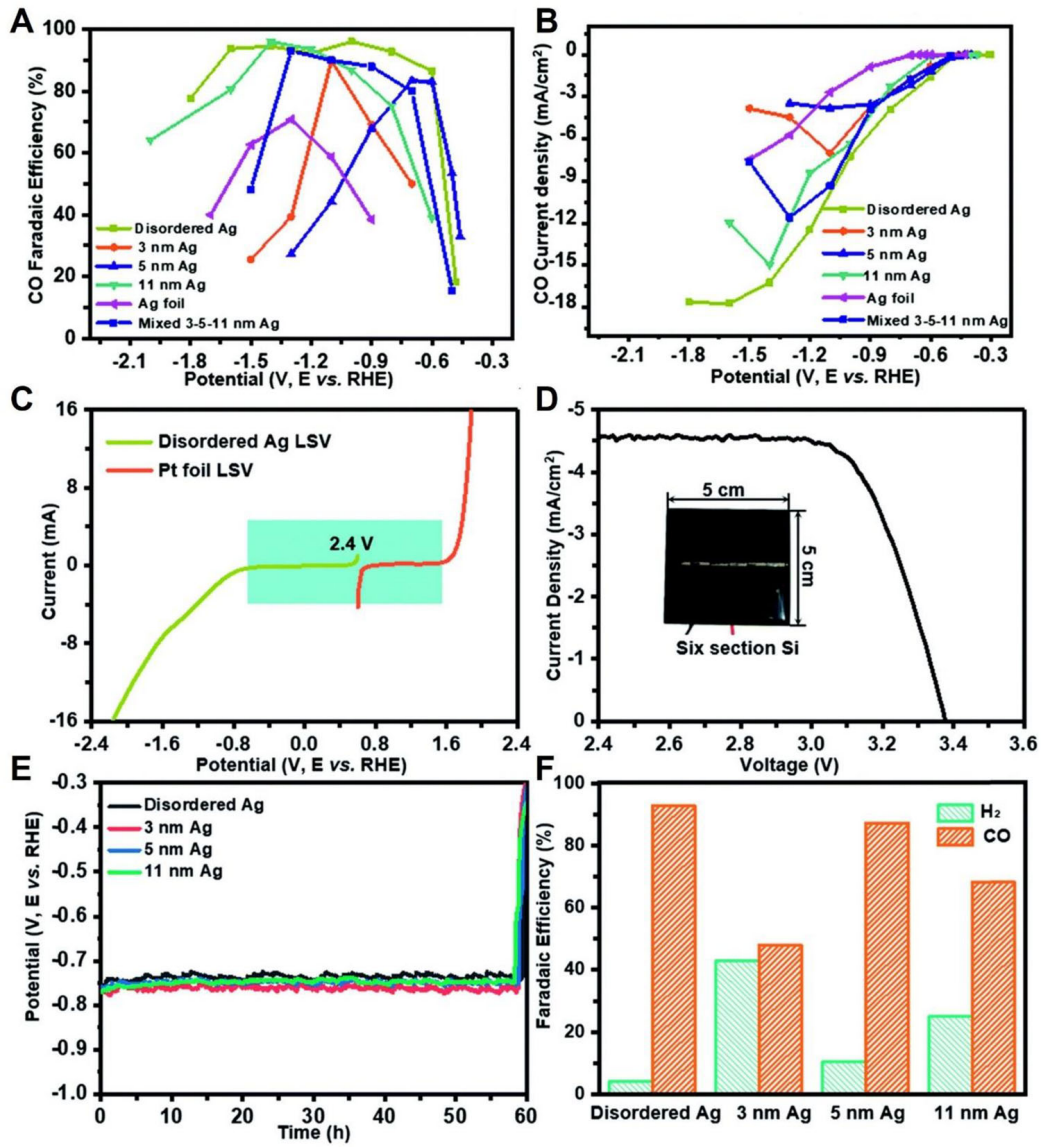
PV‐EC performance of CO_2_RR. (A) FE toward CO in CO_2_ saturated 0.1 m KHCO_3_ and (B) CO partial current density (*j*_co_) depending over a broad applied potential. (C) The linear sweep voltammetry (LSV) curve of Ag (CO_2_RR) and Pt film (HER) at a scan rate of 50 mV s^−1^. (D) The *I/V* curve shows the photovoltaic performance of a six‐section a‐Si cell under AM 1.5 G illumination. (E) Cathode voltage of chronoamperometry measurement (F) and FE toward CO in the PV‐EC device characteristic assisted by a six‐section a‐Si photovoltaics. Reproduced with permission.^[^ ^111^ ^]^ Copyright 2018, Royal Society of Chemistry.

**FIGURE 6:**
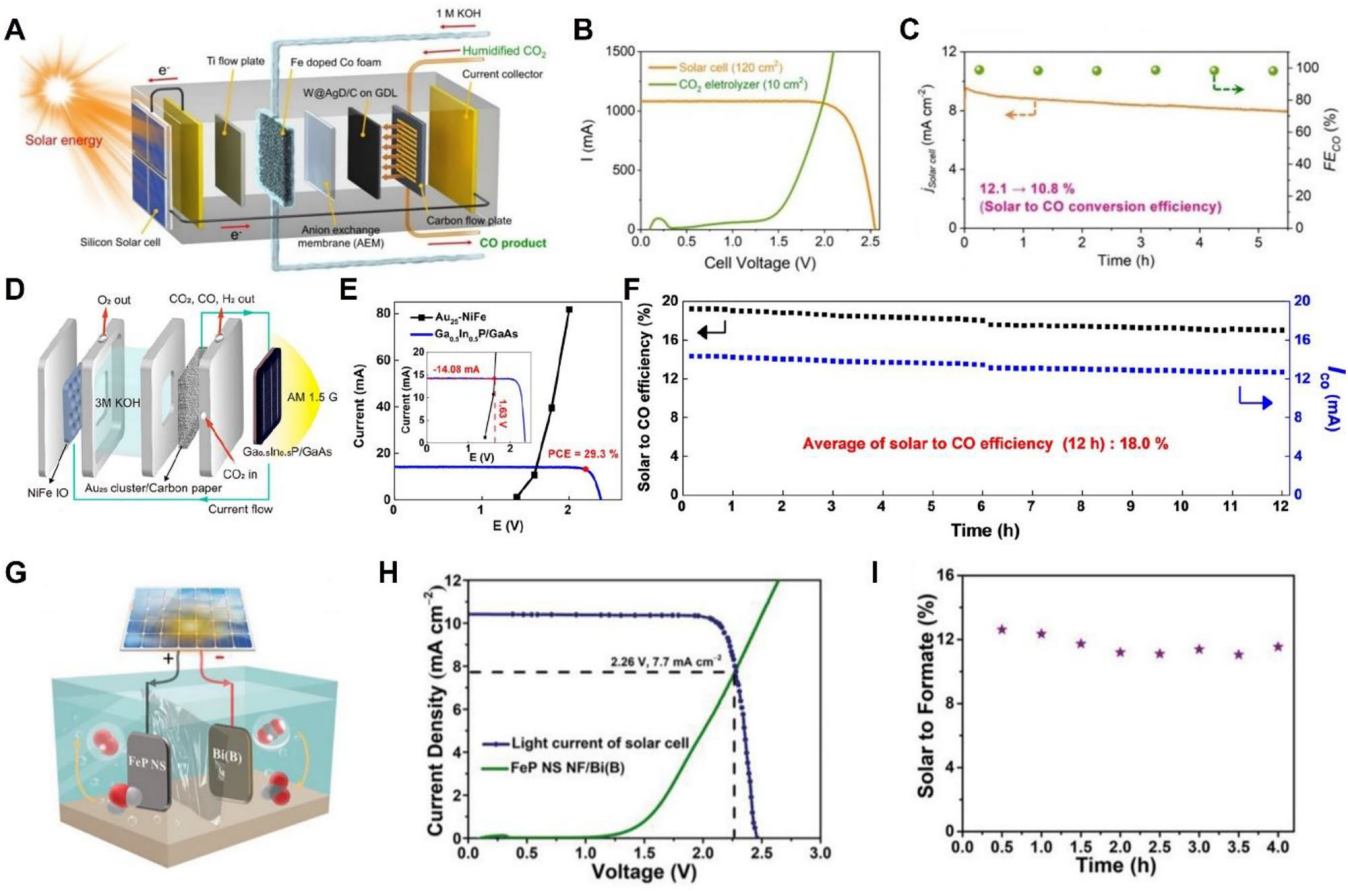
PV‐EC performance of CO_2_RR. (A) Schematic of the PV‐EC system. PV‐EC devices are composed of W@AgD/C on 10 cm^2^ GDL as cathode, 3–6 silicon cells in series as modules with a size of 10 cm × 12 cm, Fe‐doped Co foam as an anode, and anion exchange membrane. (B) Current density over an applied cell voltage (green) from the *I–V* curve and (C) FE toward CO of photovoltaics‐assisted CO_2_RR system under AM 1.5 G (orange). Reproduced with permission.^[^ ^114^ ^]^ Copyright 2021, Elsevier. (D) Schematic illustration of the PV‐EC device. PV‐EC CO_2_RR system is composed of Ga_0.5_In_0.5_P/GaAs tandem structure as solar cells, Au_25_ cluster as cathode part, NiFe inverse opal as an anode, and 3 m KOH as electrolyte. (E) *I–V* curves of the Ga_0.5_In_0.5_P/GaAs tandem photovoltaics and Au_25_‐NiFe under AM 1.5G. (F) STC efficiency of solar‐driven CO_2_RR during 12 h. Reproduced with permission.^[^ ^115^ ^]^ Copyright 2020, American Chemical Society. (G) Schematic of the PV‐EC device. Bi(B) and FeP nanosheets supported on Ni foam were utilized as cathode and anode, respectively, for PV‐EC system with GaInP/GaInAs/Ge solar cell. (H) *J–V* curves under AM 1.5 G and (I) STF efficiency during 4 h of PV‐CO_2_RR system. Reproduced with permission.^[^ ^116^ ^]^ Copyright 2021, John Wiley and Sons.

Disordered Ag nanoparticles were used as the cathode to improve the selectivity and durability for CO_2_ to CO formation, Pt foil was employed as the anode, and electrical energy was provided by a six‐section an‐Si PV cell.^[^ ^111^ ^]^ The SEM images show that the disordered Ag nanoparticles have an irregular size distribution, while the 3, 5, and 11 nm of Ag on the carbon support are uniform in size. To confirm the FE_CO_ and j_CO_, the CO_2_RR performance of Ag nanoparticles was evaluated in an H‐cell filled with a 0.1 m KHCO_3_ electrolyte. While a maximum FE_CO_ of 83%, 90%, and 95% were confirmed on 3, 5, and 11 nm of Ag nanoparticles, respectively, disordered Ag exhibited FE_CO_ of more than 90% over a wide range of potentials −0.6 to −1.7 V versus RHE (Figure 5A). In addition, the disordered Ag nanoparticles had *j*_CO_ of −16.7 mA cm^−2^ higher than other uniform Ag nanoparticles at −1.8 V versus RHE (Figure 5B). In addition, linear sweep voltage (LSV) measurements on disordered Ag were performed on CO_2_RR at the cathode and OER at the anode to determine the required light‐inducing voltage for driving the PV‐EC system. The results indicated that a voltage of 2.4 V was required (Figure 5C). Six‐section a‐silicon‐PV, which has an area of 25 cm^2^ providing 3.38 V of circuit voltage, was combined with the EC system to confirm the electrocatalytic performance of PV‐EC (Figure 5D). As shown in Figure 5E, during the PV‐EC system test, a potential of 0.75 V was observed at the cathode electrode. Owing to the active proton‐electron coupling transfer (PECT) process on disordered Ag, the selectivity of PV‐EC performance exhibited an FE_CO_ of 92.7%, which is significantly higher than other uniform Ag catalysts (Figure 5F). Thus, owing to the excellent electrocatalytic properties with an appropriate Tafel slope (128 mV dec^−1^) and overpotential, disordered Ag noble metals show high STC efficiency when combined with a‐Si‐PV.^[^ ^112^, ^113^ ^]^ Another promising CO_2_RR catalyst, which has high efficiency in CO formation when combined with a PV system, is Au, which is a noble metal. For example, Wang et al. fabricated needle‐like nano‐Au on carbon paper using a one‐step electrodeposition method as a cathode for efficient CO_2_RR and nanosheet‐like NiFe hydroxide on Ni foam via a hydrothermal method as an anode for oxygen evolution.^[^ ^37^ ^]^ Needle‐like nano‐Au exhibited excellent electrochemical CO_2_RR performance through low onset overpotential of less than 160 mV, a low Tafel slope of 47 mV dec^−1^, and a maximum FE_CO_ of ≈92% at −0.57 versus RHE. To further explain the Tafel value, catalysts with low Tafel slopes indicate that the initial rate‐determining chemical step is *COOH formation by facilitating the equilibrium state for the adsorbed CO_2_^·–^ intermediate. To build a complete PV‐powered EC system efficiently, GaAs (InGaP/GaAs/Ge) was adopted, recoding a high photoconversion rate of 37.9% and stable durability of 24 h with an average FE_CO_ of 92% in a CO_2_ saturated 0.5 m KHCO_3_ electrolyte under continuous electrolysis. Lee et al. reported a carbon‐supported tungsten‐seed‐based 3D silver dendrite (W@AgD) as a CO_2_RR catalyst for CO formation, by investigating a zero‐gap CO_2_ electrolyzer.^[^ ^114^ ^]^ As shown in Figure 6A of the scheme of the STC system, to compose a complete PV‐EC system, 3−6 silicon solar cells were assembled in series as modules with a size of 10 cm × 12 cm, and as the OER catalyst in charge of the anode part, Fe‐doped Co foam, which exhibited high catalytic activity in alkaline media, was used. The assembled PV‐EC system varied the number of silicon‐based solar cells to confirm the optimized *I–V* curves and exhibited a high STC conversion rate of 12.1% with a current of 1.1 A under AM 1.5 G, which is close to the highest value among silicon‐based PV‐EC systems, and also exhibited excellent FE_CO_ of 95% (Figure 6B,C). Kim et al. reported another excellent PV‐EC system that is advantageous for generating CO. Their system consisted of an Au_25_ cluster placed on carbon paper and was used as a cathode, NiFe inverse opal was used as an anode, and Ga_0.5_In_0.5_P/GaAs tandem PV cell was the serving solar energy (Figure 6D).^[^ ^115^ ^]^ As displayed in Figure 6E, the *I–V* characteristics of the individual series‐connected Ga_0.5_In_0.5_P/GaAs tandem cell and CO_2_ electrolyzer with Au_25_ cluster, were confirmed to match the interaction of two curves at −14 mA at 1.63 V. Moreover, the PV‐EC system combined with the Au_25_ cluster, NiFe inverse opal, and tandem solar cells exhibited an excellent average of solar to CO efficiency of 18% under continuous reaction for 12 h (Figure 6F). To attain a high STF efficiency, Chen et al. fabricated boron‐doped bismuth (Bi(B)) by anticipating the unique electronic properties of Bi(B) that regulate the free energy of the OCHO* intermediate by inducing the movement of the p‐electron state to the Fermi level.^[^ ^116^ ^]^ As shown in Figure 6G, the STF efficiency was evaluated using Bi(B) as an efficient CO_2_ catalyst and FeP nanosheets supported on Ni foam as an OER catalyst with commercial GaInP/GaInAs/Ge solar cells for efficient PV‐EC devices. As a result, this PV‐EC system achieved the best record of STC of 11.8%, accompanied by high FE for formate of 93% under the CO_2_RR system (Figure 6H,I).

### 4.2 C_2+_ product

**FIGURE 7:**
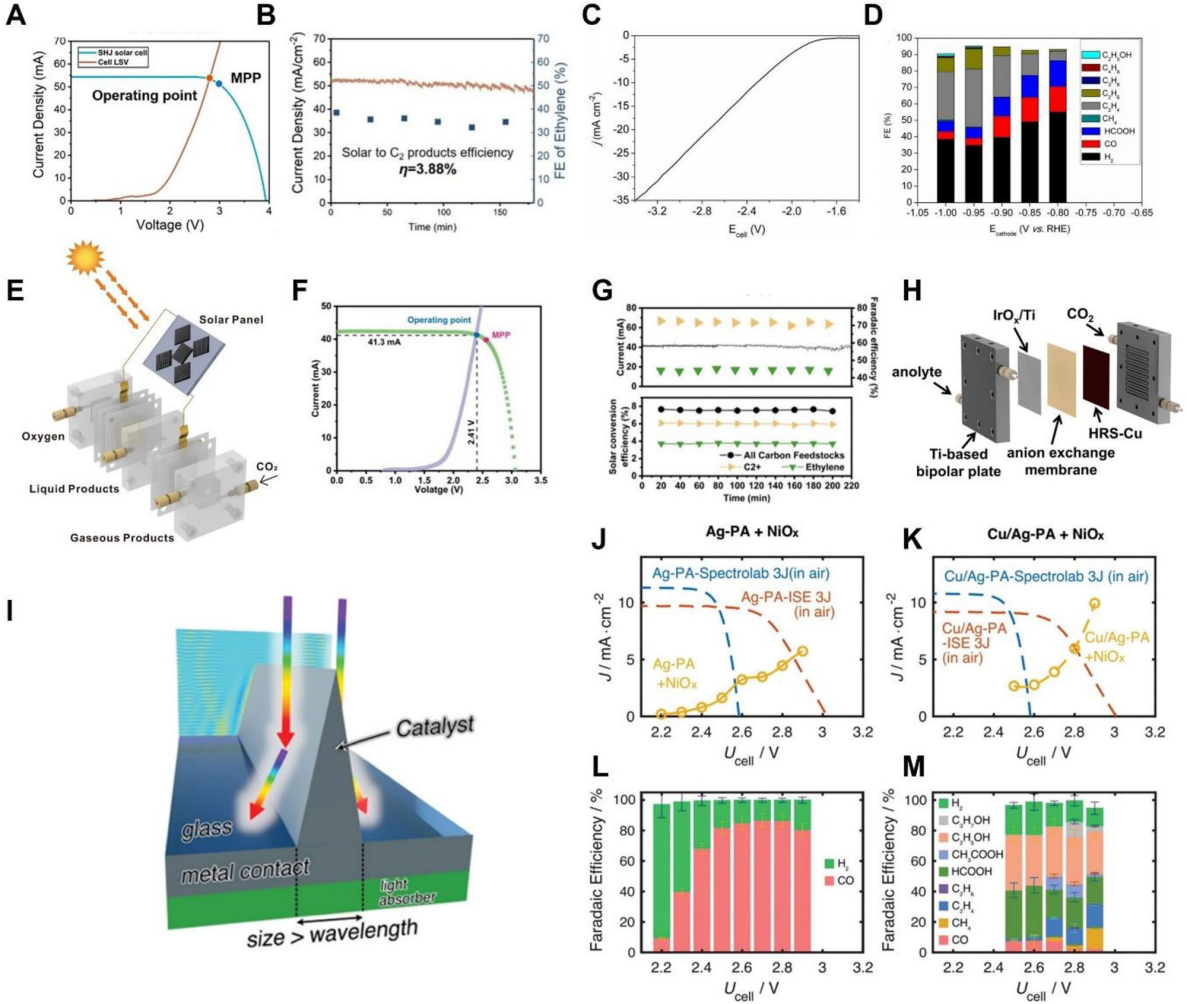
PV‐EC performance of CO_2_RR. (A) From the *J–V* characteristic, the intersection of the six‐series a‐Si/c‐Si heterojunction (SHJ) module and the operating current of the EC cell (brown) under AM 1.5 G (blue) is represented. (B) The long‐term stability test of solar‐assisted CO_2_RR system over 150 min. Reproduced with permission.^[^ ^129^ ^]^ Copyright 2020, American Chemical Society. (C) *J–V* characteristic of the EC cell and (D) FE toward diverse carbon compounds of 1 cm^2^ DN‐CuO electrodes as cathode part at broad applied potentials. Reproduced with permission.^[^ ^16^ ^]^ Copyright 2019, National Academy of Sciences. (E) Schematic illustration of the solar‐driven CO_2_RR device. (F) *I–V* curves of the PV‐EC device composed of HRS‐Cu as cathode and Si photodiode as the solar energy absorbers. *I–V* curves consist of the photovoltaic (green), electrocatalytic (purple), operating point marked by a blue dot, and MPP marked by a red dot. (G) FE toward ethylene and C_2+_ value‐added carbon compound and solar‐to‐electricity conversion value of the photovoltaic‐driven CO_2_RR system. (H) Schematic illustration of scale‐up PV‐EC device composed of MEA and enlarged 4 cm^2^ and 25 cm^2^ of cathode electrodes. Reproduced with permission.^[^ ^130^ ^]^ Copyright 2021, Springer Nature. (I) Schematic design of triangular Ag metal PA connected to the top layer of 3J III–V semiconductor photoabsorber. *J–V* curves and FE toward carbon compounds of (J,L) Ag‐PA + NiO*_x_* and (K,M) Cu/Ag‐PA +NiO*_x_* in CO_2_‐saturated 0.1 m KHCO_3_ electrolyte. Reproduced with permission.^[^ ^131^ ^]^ Copyright 2022, John Wiley and Sons.

By coupling with PV, an EC system consisting of Cu electrocatalysts is also utilized to efficiently reduce CO_2_ for C_2+_ value‐added hydrocarbons. Si (series) solar cells,^[^ ^117^ ^]^ dye‐sensitized solar cell (DSSC) solar cells,^[^ ^118^ ^]^ copper‐indium‐gallium‐selenide (CIGS) solar cells,^[^ ^119^ ^]^ and perovskite solar cells^[^ ^16^ ^]^ are some of the solar cell types that are used to provide insufficient energy to PV‐integrated EC systems using Cu‐based electrocatalysts as a cathode.^[^ ^120^ ^]^ However, the conversion efficiency to C_2+_ using Cu still faces limitations compared to C_1_ selectivity of over 90%. Therefore, to improve C_2+_ formation, various methods, including exposed facet,^[^ ^121^, ^122^, ^123^ ^]^ size effect,^[^ ^124^ ^]^ morphology change,^[^ ^125^ ^]^ defects,^[^ ^126^ ^]^ oxide state manipulation,^[^ ^127^ ^]^ and grain boundaries^[^ ^128^ ^]^ are explored from many perspectives. In addition, we discuss the ideal PV‐EC in combination with an efficient Cu catalyst fabricated by the above strategies. Chen et al. reported a grain‐boundary‐rich Cu, which was fabricated by controlling the grain growth of Cu via electrodeposition, as an efficient PV‐EC CO_2_RR electrocatalyst and achieved a high solar‐to‐C_2+_ conversion efficiency (STC).^[^ ^129^ ^]^ In electrochemical performance, grain‐boundary‐rich Cu (GB‐Cu) exhibited an exceptional FE of 73% for C_2+_ formation (propanol, ethylene, and ethanol) over a wide range of potentials, in particular, FE of 31.74% for ethanol was confirmed at a high current density of 45 mA cm^−2^ at −1.3 V versus RHE. An assembled PV‐EC system, which was composed of GB‐Cu and Se‐(NiCo)S*_x_*/(OH)*_x_* nanosheets as the cathode and anode, respectively, using a six‐series a‐Si/c‐Si heterojunction (SHJ) module as the photocathode, showed FE of 68% for C_2+_ formation and STC conversion efficiency of 3.88%, accompanied by well‐matched LSV curves of each PV and EC system (Figure 7A,B). Huan et al. adopted an oxide‐derived strategy for both cathode and anode catalysts, in which dendritic nanostructured Cu oxide (DN‐CuO) with efficient mass transfer by lowering mass transport losses was used to limit the poisoning of the cathode electrode.^[^ ^16^ ^]^ As illustrated in Figure 7C,D, an electrochemical cell using DN‐CuO as both electrodes exhibited low electrolyte resistance at a high current density (25 mA cm^−2^) at a cell potential below 3 V from the LSV curve and yielded a high production rate from FE toward C_2+_ formation. Zhang et al. achieved a maximum FE of 58.6% for ethylene by fabricating Cu (100)‐rich films reducing the energy barrier of C‐C coupling formation using the dynamic deposition‐etch‐bombardment method and further applied the Cu (100)‐rich films to the efficient cathode portion of the PV‐EC system.^[^ ^130^ ^]^ As illustrated in Figure 7E, a solar‐driven electrochemical CO_2_RR system was constructed, where high‐power reactively sputtered Cu films (HRS‐Cu) were used as the cathode, and a Si photodiode was used as the solar energy absorber. The *I*–*V* characteristic of the PV‐EC system revealed an intersection point between the photovoltaic and electrocatalytic curves at an operating current density of 41.3 mA and a voltage of 2.41 V under simulated AM 1.5G illumination. This intersection point corresponds to the maximum power point (MPP) of the solar panel, demonstrating the efficient solar‐to‐electricity conversion capabilities of the PV‐EC system (Figure 7F). As shown in Figure 7G, total FE of ≈72% for C_2+_, ethylene of ≈45%, and STC efficiency of ≈6% with 40 mA of current were confirmed by chronoamperometry measurements under simulated AM 1.5G illumination for 220 min. In addition, to scale up the PV‐EC system, a membrane electrode assembly system (MEA), which has advantages such as no requirement for additional catalyst loading steps, no electrode contamination, and suitability for large‐area electrodes, was adopted (Figure 7H). When the cathode electrode was enlarged to 4 cm^2^ and 25 cm^2^, the current density and maximum FE for ethylene reached 120 mA cm^−2^ and 58.6%, and 480 mA cm^−2^ and 50.9%, respectively. Ideal PV‐EC, which was reported by Cheng et al., composed of selective electrodeposition of Cu catalysts on Ag catalyst prisms, covered with an optimal amount (35%) of surface area, exhibits excellent stability.^[^ ^131^ ^]^ As shown in Figure 7I, a semitransparent metal prism array (PA) was connected to the top layer of triple junction (3J) III–V semiconductors to suppress hydrogen evolution and achieve efficient light harvesting. The intersections between photovoltaics, including the Spectrolab stack, which is the light‐limiting current in the middle cell, and FhG‐ISE 3J, which is the light‐limiting current in the bottom cell, and electrocatalysts such as Ag‐PA and Cu/Ag‐PA with NiO*_x_* as an anode, are displayed from *J–V* measurements in the 0.1 m CO_2_‐purged KHCO_3_ (Figure 7J,K). By analyzing the *J*–*V* curves, the intersection between Ag‐PA+NiO*_x_* and Ag‐PA‐Spectrolab 3J, as well as Ag‐PA‐ISE 3J, was identified, confirming a high FE of ≈80% for CO at broad cell voltages (2.5–2.9 V) and a cell voltage (*U*_cell_) of 2.56 V and current (*J*) of 2.65 mA cm^−2^ for Ag‐PA‐Spectrolab 3J and *U*_cell_ of 2.85 V and *J* of 5.13 mA cm^−2^ for Ag‐PA‐ISE 3J (Figure 7I,L). The addition of electrodeposited Cu on Ag‐PA resulted in a *J*–*V* intersection displaying a *U*_cell_ of 2.56 V and *J* of 2.60 for Spectrolab 3J, and *U*_cell_ of 2.8 V and *J* of 5.97 mA cm^−2^ for Ag‐PA‐ISE 3J (Figure 7K). Furthermore, Cu/Ag‐PA exhibited an FE of ≈30% for C_2_H_5_OH in the voltage range of 2.5–2.9 V, as well as the formation of value‐added C_2+_ carbon compounds (Figure 7M).

## 5 PHOTOVOLTAIC‐POWERED PHOTOELECTROCATALYTIC CO_2_RR REACTOR

The PV‐PEC system utilizes a single‐junction or multi‐junction PV and a photoelectrode to convert light energy into voltage and current, which drives a redox reaction at the electrode interface.^[^ ^132^, ^133^ ^]^ Compared to other CO_2_ reduction technologies, PV‐PEC systems have the potential to achieve higher efficiencies by utilizing both electricity and photons generated by the photovoltaic. Furthermore, the system can operate under various conditions and can be tailored to produce specific chemical products based on the choice of catalyst. The device‐activity relationships of PV‐PEC system are multifaceted and influenced by a variety of factors, including the PV material properties, the catalyst's activity and selectivity, and the system's operating conditions such as temperature, pressure, and electrolyte composition. Given, this section intends to provide a comprehensive account of PV‐PEC system, encompassing different types of catalysts, system configurations, and environmental factors.

**FIGURE 8:**
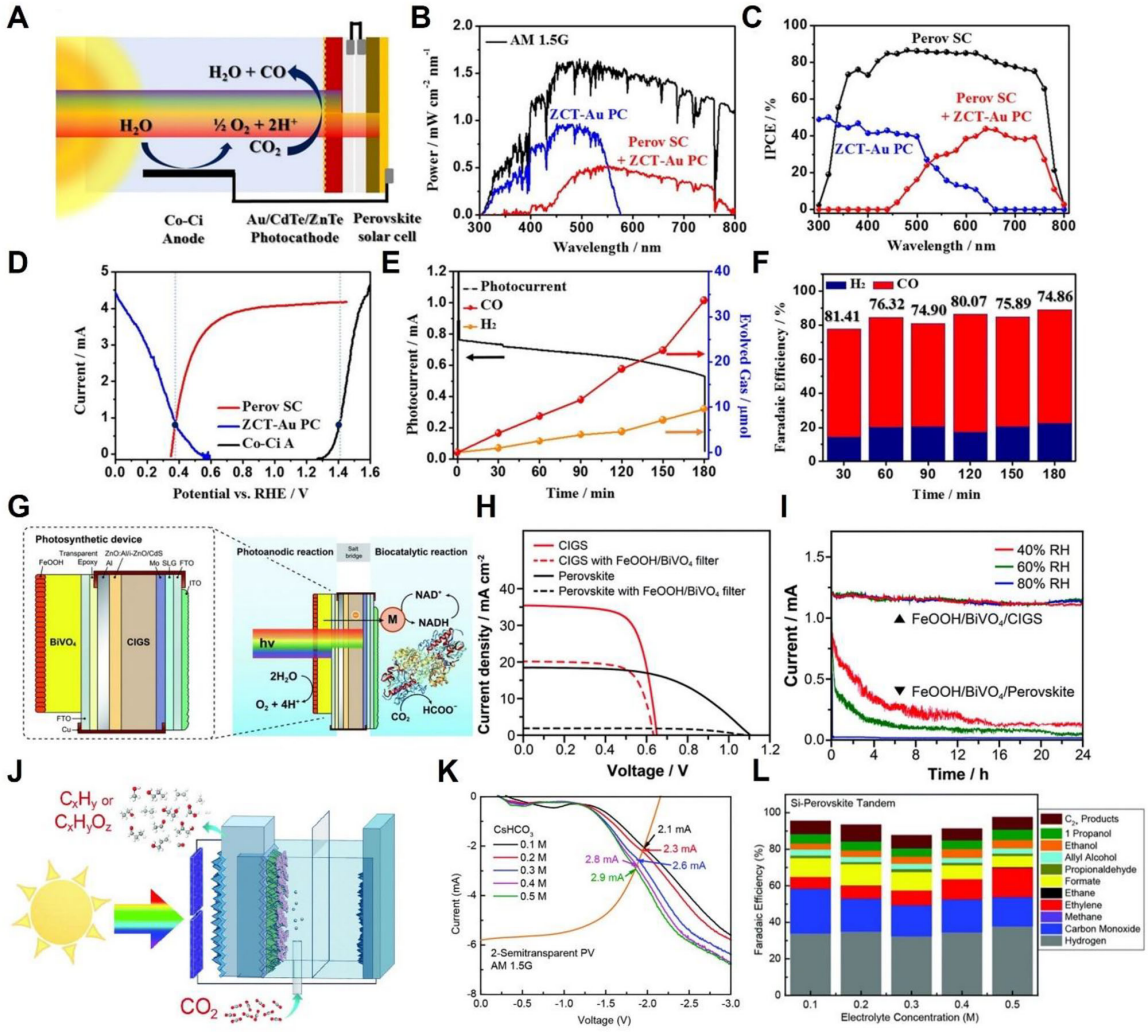
PV‐PEC performance of CO_2_RR. (A) Schematic design of the stacked tandem structure of bias‐free PV‐PEC system comprising a gold‐decorated triple‐layer ZnO@ZnTe@CdTe core–shell nanorod array photocathode, CH_3_NH_3_PbI_3_ perovskite solar cell, and Co–Ci anode. (B) Absorption property of two light absorbers under AM 1.5G. (C) Plots of incident photon‐to‐current conversion efficiency (IPCE) of ZCT‐Au‐PC, Perov SC, and Perov SC + ZCT‐Au PC. (D) *J–V* characteristic of PV‐PEC in the stacked tandem structure composed of ZCT‐Au photocathode and Co–Ci anode. (E) Generated gas production and photocurrent measured by chronoamperometry for 3 h of bias‐free PV‐PEC device. (F) FEs toward H_2_ and CO of the stacked tandem structure of bias‐free PV‐PEC device measured for 3 h of CO_2_RR in CO_2_‐purged KHCO_3_ solution under 1 sun illumination. Reproduced with permission. ^[^ ^134^ ^]^ Copyright 2016, American Chemical Society. (G) Schematic diagram of assembled FeOOH/BiVO_4_/CIGS tandem device of unbiased PV‐PEC system for formate conversion. (H) Comparison of *J–V* profiles of CIGS and perovskite solar absorber with/without FeOOH/BiVO_4_ filtered light. (I) Chronoamperometry measurement of FeOOH/BiVO_4_/CIGS and FeOOH/BiVO_4_/Perovskite depending on relative humidity (RH) values. Reproduced with permission.^[^ ^41^ ^]^ Copyright 2012, Royal Society of Chemistry. (J) Schematic diagram of PV‐PEC system comprising an Ag‐supported dendritic Cu as Si‐photocathode, IrO_2_ nanotube anode, with two series‐connected semi‐transparent halide perovskite solar cells. (K) *J–V* curves of the Si‐photocathode (bottom side) and perovskite PV cell (top side) depending on the concentrations of the CsHCO_3_ electrolyte under 1 sun illumination. (L) FEs toward value‐added carbon compound depending on the concentrations of the CsHCO_3_ electrolyte of unbiased Si‐perovskite tandem PV‐PEC device under 1 sun illumination. Reproduced with permission.^[^ ^135^ ^]^ Copyright 2019, Royal Society of Chemistry.

As shown in Figure 8A, Jang et al. designed the stacked tandem cell structure of a PV‐PEC system for efficient STC conversion efficiency from a photoelectrode, which has excellent light harvesting of higher‐energy photons, and from a single junction of perovskite PV, which has lower‐energy photons.^[^ ^134^ ^]^ In a concrete structure, the gold‐decorated triple‐layer ZnO@ZnTe@CdTe (ZCT) core–shell nanorod array with facilitated charge separation and a narrow band gap with excellent catalytic efficiency was utilized as a photocathode, a CH_3_NH_3_PbI_3_ perovskite solar cell in tandem was adopted for efficient light harvesting, and Co–Ci was situated in a light‐blocked place as an OER anode. The absorption property, including incident photon‐to‐current conversion efficiency (IPCE), of two light absorbers under AM 1.5 G indicated that while the power density of Au nanoparticles decorated a ZCT (ZCT NR‐Au) photocathode was reduced to 55%, accompanied by unchanged open‐circuit voltage, most of light it used was below 550 nm, as shown in Figure 8B,C. From the *J–V* characteristic, the operating point at the current of 0.85 mA was confirmed by the intersection of the perovskite solar cell and the photocathode in an unbiased tandem device for spontaneous photoelectrochemical CO_2_RR (Figure 8D). Furthermore, the evolution of gaseous products containing CO and H_2_ was measured for 3 h under 1 sun illumination by chronoamperometry with unbiased external voltage, and a ZnO@ZnTe@CdTe‐Au photocathode with single‐junction perovskite achieved FE of 74.9% for CO in the CO_2_‐purged KHCO_3_, which shows excellent selectivity among induced corrosion of Te‐based materials (Figure 8E,F). Similarly, the production of value‐added products with tandem structures combined with solar cells and Cu‐based photocathodes has attracted considerable attention. For the effective formation of HCOOH, one of the valuable C_1_ products, Kim et al. introduced assembled FeOOH/BiVO_4_/CIGS tandem devices that do not require external bias such as 1.2 V.^[^ ^41^ ^]^ For the assembled tandem device, a single Cu(In,Ga)Se_2_ (CIGS) solar absorber was utilized as a PV cell owing to its photo advantages such as adequate direct band gap (1.12 eV) and excellent durability in an aqueous electrolyte under the CO_2_RR system (Figure 8G). In addition, FeOOH/BiVO_4_ adjusted in thickness retained high stability and optimal current as a photoanode situated at the top cell, and a mesoporous indium tin oxide (*meso*ITO) cathode was used as the working electrode. As shown in Figure 8H,I, photoelectrochemical profiles including *J–V* characteristics and chronoamperometry measurement of the combined CIGS‐based tandem device compared to perovskite solar cell exhibited improved photoelectrochemical performance in the following metrics: an open‐circuit voltage (0.64 V), short‐circuit current density (35.68 mA cm^−2^), a fill factor (65%), and power conversion efficiency (15.01%). In contrast to the humidity‐sensitive perovskite‐based tandem cells showing formate formation below 5 mm under relative humidity (RH) environments, FOOH/BiVO_4_/CIGS/*meso*ITO CIGS‐based system produced ≈6 mm of formate concentration regardless of RH environment value of more than 80%. For efficient CO_2_RR toward multi‐carbon (C_2+_) formation by photovoltaic‐biased photoelectrocatalysis, Gurudayal et al. designed a PV‐PEC device utilizing an Ag‐supported dendritic Cu as Si‐photocathode (bottom photoabsorber).^[^ ^135^ ^]^ A tandem system consisting of IrO_2_ nanotube anode and two series‐connected semi‐transparent perovskite solar cells with a band gap of 1.58 eV was tested in 0.1 m CO_2_‐purged CsHCO_3_ under 1 sun illumination, as shown in Figure 8J. The *J*–*V* curve analysis of the photocathode and two series‐connected PV intersections confirmed operating currents ranging from 2.1 to 2.9 mA, depending on the concentration of the CsHCO_3_ electrolyte, as seen in Figure 8K. The outstanding FE of the system for diverse carbon compounds by suppressing HER evolution and STC efficiency of 3.5% with unbiased external voltage are depicted in Figure 8L. The use of perovskite solar cells, which are cost‐efficient, results in high STC efficiency by combining tandem devices with an efficient photocathode. The PV‐EC and PV‐PEC devices are listed in Table 3.

**TABLE 3:** Summary of PV‐EC and PV‐PEC devices for CO_2_RR.

PV‐assisted CO_2_RR device	CO_2_RR catalyst	OER catalyst	Photovoltaics	Product	FE (%)	Solar‐to‐energy (%)	Ref.
PV‐EC	Disordered Ag	Pt‐foil	Si	CO	92.8%	≈0.1% (Solar‐to‐CO)	^[^ ^111^ ^]^
PV‐EC	Needle‐like nano‐Au	Nanosheet‐like NiFe hydroxide	GaAs (InGaP/GaAs/Ge)	CO	92%	15.6% (Solar‐to‐CO)	^[^ ^37^ ^]^
PV‐EC	W@AgD	Fe‐doped Co foam	Si	CO	95%	12.1% (Solar‐to‐CO)	^[^ ^114^ ^]^
PV‐EC	Au_25_ cluster	NiFe inverse opal	Ga_0.5_In_0.5_P/GaAs	CO	90%	18% (Solar‐to‐CO)	^[^ ^115^ ^]^
PV‐EC	Boron‐doped bismuth (Bi(B))	FeP nanosheet	GaInP/GaInAs/Ge	Formate	93%	11.8% (Solar‐to‐formate)	^[^ ^116^ ^]^
PV‐EC	Oxide‐derived Cu	In_2_O_3_	Si	C_2_H_4_	31.9%	2.9% (Solar‐to‐fuel)	^[^ ^117^ ^]^
PV‐EC	Grain‐boundary‐rich Cu	Se‐(NiCo)S*_x_*/(OH)*_x_* nanosheets	a‐Si/c‐Si heterojunction	C_2_H_5_OH	31.7%	3.8% (Solar‐to‐C_2+_)	^[^ ^129^ ^]^
PV‐EC	Dendritic nanostructured CuO (DN‐CuO)	Dendritic nanostructured CuO (DN‐CuO)	Perovskite	C_2_H_4_	34%	2.3% (Solar‐to‐hydrocarbon)	^[^ ^16^ ^]^
PV‐EC	High‐power reactively sputtered Cu films (HRS‐Cu)	Ni foam	Si	C_2_H_4_	45%	≈4% (Solar‐to‐ethylene)	^[^ ^130^ ^]^
PV‐EC	Cu/Ag‐PA‐FhG‐ISE 3J	NiO*_x_*	III–V semiconductors	C_2_H_5_OH	≈15%	5% (Solar‐to‐fuels)	^[^ ^131^ ^]^
PV‐PEC	Pulse‐oxidized Au	CoO*_x_*	a‐Si	CO	>95%	2% (Solar‐to‐CO)	^[^ ^132^ ^]^
PV‐PEC	ZnO@ZnTe@CdTe core–shell nanorod	Co–Ci	Perovskite	CO	74.9%	0.43% (Solar‐to‐fuels)	^[^ ^134^ ^]^
PV‐PEC	MesoITO	FeOOH/BiVO_4_	CIGS	Formate	5.6 mm	0.03% (Solar‐to‐formate)	^[^ ^41^ ^]^
PV‐PEC	Ag‐supported dendritic Cu	IrO_2_ nanotube	Perovskite	CO	>20%	3.5% (Solar‐to‐chemical)	^[^ ^135^ ^]^

## 6 CONCLUSIONS AND PERSPECTIVE

In this review, we introduce diverse systems, including electrochemical (EC), photoelectrochemical (PEC), photovoltaic‐assisted electrochemical (PV‐EC), and photovoltaic‐assisted photoelectrochemical (PV‐PEC), for efficient CO_2_ reduction and conversion to achieve carbon neutrality by introducing catalysts, photoabsorbers, and ideal bias‐free PV‐assisted devices. In the EC system, Cu, the only metal capable of producing high value‐added C_2+_ carbon compounds, has limitations due to the lack of energy supply for the formation of C─C couplings, while electrocatalysts such as noble metals and MOF‐based and single atom‐based materials show excellent CO_2_ conversion rates of approximately 100% for C_1_ products. Therefore, as mentioned, designing Cu‐based materials that establish various strategies, such as manipulating surfaces, reconfiguring morphology, and inducing synergies from heterogeneous catalysts, is imperative. Despite numerous efforts, the low FE toward C_2+_ chemicals is a problem to be solved from various perspectives, including not only the EC system but also PEC and PV‐PEC devices. Moreover, obstacles to the PEC system, such as complex reaction paths, large photovoltage requirements, low solar‐to‐fuel efficiency, and poor light‐harvesting properties, can be overcome by combining a co‐catalyst with a light‐absorbing semiconductor. Additionally, as in the EC system, manufacturing ideal PEC catalysts causing synergetic effects from heterogeneous materials and coating photocathodes with MOF materials may be a potential substitute for expensive noble metal catalysts, maintaining high energy conversion efficiency.

To acquire large‐scale installations of CO_2_RR devices, we proposed two novel PV‐powered systems: PV‐EC and PV‐PEC. Both systems have an outstanding ability to lead the techno‐economy to achieve high STC efficiency by serving the 2.6 V needed to reduce CO_2_ from photovoltaics. Despite various technological advancements, high energy consumption and inefficient processes, as indicated by large voltage requirements derived from the analysis of MPP and *I/J–V* characteristics, remain significant challenges in the field. To overcome these challenges, Si‐based, perovskite‐based, DSSC‐based, CIGS‐based, and GaAs‐based solar absorbers, as well as those of multi‐junction or tandem structures, have gained a lot of attention. Nevertheless, Group III–V semiconductors composed of multi‐component elements containing GaAs‐based absorbers show excellent STC efficiency. However, they are not cost‐efficient in constructing a light‐harvesting system. On the other hand, perovskite solar cells connected in series, which have tunable band gaps, high STC efficiency, high open‐circuit voltages, and cost efficiency, exhibit a lack of long‐term stability in the CO_2_RR system. In order to attain the optimal methodology, the PV‐PEC apparatus must function by harnessing voltage from both the photocathode and PV cell, thereby necessitating a lower voltage than that required by the PV‐EC, while simultaneously generating a heightened current density that culminates in a superior STC efficiency. Furthermore, a concomitant need for progress in materials science, catalyst design, system integration, and process engineering is also paramount. Overall, to realize bias‐free PV‐EC and PV‐PEC devices, high‐performance of various combinations of cathodes and anode catalysts, zero‐gap electrolysis, and engineering series‐connected photovoltaics are required, and further development is essential for industrial applications.

## CONFLICT OF INTEREST STATEMENT

The authors declare no conflicts of interest.
